# Estimation and Optimization of Composite Outcomes

**Published:** 2021-01

**Authors:** Daniel J. Luckett, Eric B. Laber, Siyeon Kim, Michael R. Kosorok

**Affiliations:** Genospace, Boston, MA 02108, USA; Department of Statistics, North Carolina State University, Raleigh, NC 27607, USA; Department of Biostatistics, University of North Carolina at Chapel Hill, Chapel Hill, NC 27607, USA; Departments of Biostatistics and Statistics & Operations Research, University of North Carolina at Chapel Hill, Chapel Hill, NC 27599, USA

**Keywords:** Individualized treatment rules, Inverse reinforcement learning, Precision medicine, Utility functions

## Abstract

There is tremendous interest in precision medicine as a means to improve patient outcomes by tailoring treatment to individual characteristics. An individualized treatment rule formalizes precision medicine as a map from patient information to a recommended treatment. A treatment rule is defined to be optimal if it maximizes the mean of a scalar outcome in a population of interest, e.g., symptom reduction. However, clinical and intervention scientists often seek to balance multiple and possibly competing outcomes, e.g., symptom reduction and the risk of an adverse event. One approach to precision medicine in this setting is to elicit a composite outcome which balances all competing outcomes; unfortunately, eliciting a composite outcome directly from patients is difficult without a high-quality instrument, and an expert-derived composite outcome may not account for heterogeneity in patient preferences. We propose a new paradigm for the study of precision medicine using observational data that relies solely on the assumption that clinicians are approximately (i.e., imperfectly) making decisions to maximize individual patient utility. Estimated composite outcomes are subsequently used to construct an estimator of an individualized treatment rule which maximizes the mean of patient-specific composite outcomes. The estimated composite outcomes and estimated optimal individualized treatment rule provide new insights into patient preference heterogeneity, clinician behavior, and the value of precision medicine in a given domain. We derive inference procedures for the proposed estimators under mild conditions and demonstrate their finite sample performance through a suite of simulation experiments and an illustrative application to data from a study of bipolar depression.

## 1. Introduction

Precision medicine is an approach to healthcare that involves tailoring treatment based on individual patient characteristics (Hamburg and Collins, 2010; Collins and Varmus, 2015). Accounting for heterogeneity by tailoring treatment has the potential to improve patient outcomes in many therapeutic areas. An individualized treatment rule formalizes precision medicine as a map from the space of patient covariates into the space of allowable treatments (Murphy, 2003; Robins, 2004). Almost all methods for estimating individualized treatment rules have been designed to optimize a scalar outcome (exceptions will be discussed shortly). However, in practice, clinical decision making often requires balancing trade-offs between multiple outcomes. For example, clinicians treating patients with bipolar disorder must manage both depression and mania. Antidepressants may help correct depressive episodes but may also induce manic episodes (Sachs et al., 2007; Ghaemi, 2008; Goldberg, 2008; Wu et al., 2015). We propose a novel framework for using observational data to estimate a composite outcome and the corresponding optimal individualized treatment rule.

The estimation of optimal individualized treatment rules has been studied extensively, leading to a wide range of estimators designed to suit an array of data structures and data-generating processes (Kosorok and Laber, 2019; Tsiatis et al., 2020). These estimators include: regression-based methods like Q-learning (Murphy, 2005; Qian and Murphy, 2011; Schulte et al., 2014; Laber et al., 2014a), *A*-learning (Murphy, 2003; Robins, 2004; Blatt et al., 2004; Moodie et al., 2007; Wallace and Moodie, 2015), and regret regression (Henderson et al., 2010); direct-search methods (Rubin and van der Laan, 2012; Zhang et al., 2012b; Zhao et al., 2012; Zhang et al., 2013; Zhou et al., 2017) based on inverse probability weighting (Robins, 1999; Murphy et al., 2001; van der Laan and Petersen, 2007; Robins et al., 2008); and hybrid methods (Taylor et al., 2015; Zhang et al., 2018). The preceding methods require specification of a single scalar outcome that will be used to define an optimal regime; were individual patient utilities known, then they could be used as the outcome in any of these methods. However, in general such utilities are not known though they can be elicited provided a high-quality instrument is available (Butler et al., 2018); in the absence of such an instrument, preference elicitation is difficult to apply. A method for constructing a composite utility that is best predicted using a non-parametric machine learning model is proposed by (Benkeser et al., 2020); howeer, they do not consider heterogeneous utilities or the construction of precision medicine strategies.^1^

We propose a new paradigm for estimating optimal individualized treatment rules from observational data without eliciting patient preferences. The key premise is that clinicians are attempting to act optimally with respect to each patient’s utility and thus the observed treatment decisions contain information about individual patient utilities. This idea is similar to that introduced by Wallace et al. (2018) (see also Wallace et al., 2016); however, we provide an estimator for the probability that a patient is treated optimally, rather than assuming that all patients are treated optimally. We construct estimators of individual patient utilities which do not require that clinicians are acting optimally, only that they approximately follow an optimal policy. This approach allows us to describe the goals of the decision maker and how these goals vary across patients, determine what makes a patient more or less likely to be treated optimally under standard care, and estimate the decision rule which optimizes patient-specific composite outcomes. We develop this approach in the context of a single-stage, binary decision in the presence of two outcomes. An extension to the setting with more than two outcomes is discussed in the Appendix.

Other methods for estimating optimal treatment rules in presence of multiple outcomes include using an expert-derived composite outcome for all patients (Thall et al., 2002, 2007; Murray et al., 2016; Moser et al., 2020). However, this does not account for differences in the utility function across patients and in some cases it may not be possible to elicit a high-quality composite outcome from an expert. Alternatively, multiple outcomes can be incorporated using set-valued treatment regimes (Laber et al., 2014b; Lizotte and Laber, 2016; Wu, 2016), constrained optimization (Linn et al., 2015; Laber et al., 2018; Wang et al., 2018), or inverse preference elicitation (Lizotte et al., 2012). Schnell et al. (2017) extend methods for estimating the benefiting subgroup to the case of multiple outcomes using the concept of admissibility (see also Schnell et al., 2016). However, none of these approaches provide a method for estimating an individual patient’s utility.

This work is closely related to inverse reinforcement learning (Kalman, 1964; Ng et al., 2000; Abbeel and Ng, 2004; Ratliff et al., 2006), which involves studying decisions made by an expert and constructing the utility function that is optimized by the expert’s decisions. Inverse reinforcement learning has been successfully applied in navigation (Ziebart et al., 2008) and human locomotion (Mombaur et al., 2009). Inverse reinforcement learning methods assume that decisions are made in a single environment. However, in the context of precision medicine, both the utility function and the probability of optimal treatment may vary across patients. Our approach is a version of inverse reinforcement learning with multiple environments.

This work is also related to the notion of stated and revealed preferences in the health economics literature.^2^ Viewed through this lens, our work might be characterized as using clinical decisions as a kind of surrogate for patient revealed preferences thereby avoiding the need for the elicitation of stated preferences using specialized instruments. This is advantageous as the construction of high-quality instruments is difficult and collection of preference information is not routine in many areas (Carlsson and Martinsson, 2003; Ryan et al., 2007; de Bekker-Grob et al., 2012; Soekhai et al., 2019); though see Butler et al. (2018) for an illustrative application when such an instrument is available. Challenges associated with preference elicitation for precision medicine are discussed in Laber et al. (2014b), Lizotte and Laber (2016).

In Section 2, we introduce a pseudo-likelihood method to estimate patient utility functions from observational data. In Section 3, we state a number of theoretical results pertaining to the proposed method, including consistency and inference for the maximum pseudo-likelihood estimators. Section 4 presents a series of simulation experiments used to evaluate the finite sample performance of the proposed methods. Section 5 presents an illustrative application using data from the STEP-BD bipolar disorder study. Conclusions and a discussion of future research are given in Section 6. Proofs are given in the appendix along with additional simulation results and a discussion of an extension to more than two outcomes.

## 2. Pseudo-likelihood Estimation of Utility Functions

Assume the available data are (***X***_*i*_, *A*_*i*_, *Y*_*i*_, *Z*_*i*_), *i* = 1, *…, n*, which comprise *n* independent and identically distributed copies of (***X***, *A, Y, Z*), where $X\in X\subseteq {\mathbb{R}}^{p}$ are patient covariates, A∈A={−1,1} is a binary treatment, and *Y* and *Z* are two real-valued outcomes for which higher values are more desirable. The extension to scenarios with more than two outcomes is discussed in the Appendix. An individualized treatment rule is a function d:X→A such that, under *d*, a patient presenting with covariates ***X*** = ***x*** will be assigned to treatment *d*(***x***). Let Y*(*a*) denote the potential outcome under treatment a∈A, and for any regime *d*, define $Y^{*}(d)=\sum_{a\in A}Y^{*}(a)1\{d(X)=a\}$. An optimal regime for the outcome *Y*, say $d_{Y}^{\text{opt}}$, satisfies $EY^{*}\left(d_{Y}^{\text{opt }}\right)\geq EY^{*}(d)$ for any other regime *d*. The optimal regime for the outcome *Z*, say $d_{Z}^{\text{opt}}$, is defined analogously. In order to identify these optimal regimes, and subsequently to identify the optimal regime across the class of utility functions introduced below, we make the following assumptions.

**Assumption 1** *Consistency, Y* = Y*(*A*) *and Z* = Z*(*A*).

**Assumption 2** *Positivity*, Pr(*A* = *a*|***X*** = ***x***) ≥ *c >* 0 *for some constant c and all pairs* (x,a)∈X×A.

**Assumption 3** *Ignorability*, {Y*(−1), Y*(1)}⊥*A*|***X*** *and* {Z*(−1), Z*(1)}⊥*A*|***X***.

In addition we assume that there is no interference between units nor are the multiple versions of treatment (Rubin, 1980). These assumptions are standard in causal inference (Robins, 2004; Hernan and Robins, 2010). Assumption 3 is not empirically verifiable in observational studies (Rosenbaum and Rubin, 1983; Rosenbaum, 1984).

Define $Q_{Y}(x,a)=E(Y|X=x,A=a)$. Then, under the preceding assumptions, it can be shown that $d_{Y}^{\text{opt}}(x)=\text{arg }{\text{max}}_{a\in A}Q_{Y}(x,a)$ (Zhang et al., 2012b; Qian and Murphy, 2011). Similarly, it follows that $d_{Z}^{\text{opt }}(x)=\text{arg }{\text{max}}_{a\in A}Q_{Z}(x,a)$ where $Q_{Z}(x,a)=E(Z|X=x,A=a)$. In general, $d_{Y}^{\text{opt}}(x)$ need not equal $d_{Z}^{\text{opt}}(x)$; therefore, if both *Y* and *Z* are clinically relevant, neither $d_{Y}^{\text{opt}}$ nor $d_{Z}^{\text{opt}}$ may be acceptable. We assume that there exists an unknown and possibly covariate-dependent utility *U* = *u*(*Y, Z*), where $u:{\mathbb{R}}^{2}\to \mathbb{R}$ measures the “goodness” of the outcome pair (*y, z*). The optimal regime with respect to *U*, say $d_{U}^{\text{opt}}$, satisfies $EU^{*}\left(d_{U}^{\text{opt}}\right)=Eu\left\{Y^{*}\left(d_{U}^{\text{opt}}\right),Z^{*}\left(d_{U}^{\text{opt}}\right)\right\}\geq Eu\left\{Y^{*}(d),Z^{*}(d)\right\}=EU^{*}(d)$ for any other regime *d*. The goal is to use the observed data to estimate the utility and subsequently $d_{U}^{\text{opt }}$. Define $Q_{U}(x,a)=E(U|X=x,A=a)$. For the class of utility functions we consider below, *Q*_*U*_(***x***, *a*) is a (possibly covariate-dependent) convex combination of *Q*_*Y*_ (***x***, *a*) and *Q*_*Z*_(***x***, *a*) and is therefore identifiable under the stated causal assumptions and furthermore $d_{U}^{\text{opt}}(x)=\text{arg }{\text{max}}_{a\in A}Q_{U}(x,a)$.

We assume that clinicians act with the goal of optimizing each patient’s utility and that their success in identifying the optimal treatment depends on individual patient characteristics. Therefore, we assume that the clinicians are approximately, i.e., imperfectly, assigning treatment according to $d_{U}^{\text{opt}}(x)$. If the clinician were always able to correctly identify the optimal treatment and assign $A=d_{U}^{\text{opt}}(X)$ for each patient, there would be no need to estimate the optimal treatment policy (Wallace et al., 2016). Instead, we assume that the decisions of the clinician are imperfect and that $\text{Pr}\left\{A=d_{U}^{\text{opt}}(x)|X=x\right\}=\text{expit}\left(x^{⊤}\beta \right)$ where *β* is an unknown parameter. We show in Section 2.2 that the model is identifiable under mild conditions; e.g., these exclude the possibility of a malevolent clinician that is systematically assigning poor treatments. We implicitly assume throughout that ***X*** may contain higher order terms, interactions, or basis functions constructed from patient covariates.

### 2.1 Fixed Utility

We begin by assuming that the utility function is constant across patients and takes the form *u*(*y, z*; *ω*) = *ωy* + (1 − *ω*)*z* for some *ω* ∈ [0, 1]. Lemma 1 of Butler et al. (2018) states that, for a broad class of utility functions, the optimal individualized treatment rule is equivalent to the optimal rule for a utility function of this form. Define *Q*_*ω*_(***x***, *a*) = *ωQ*_*Y*_ (***x***, *a*) + (1 − *ω*)*Q*_*Z*_(***x***, *a*) and define $d_{\omega }^{\text{opt}}(x)=\text{arg }{\text{max}}_{a\in A}Q_{\omega }(x,a)$. Let ${\hat{Q}}_{Y,n}$ and ${\hat{Q}}_{Z,n}$ denote estimators of *Q*_*Y*_ and *Q*_*Z*_ obtained from regression models fit to the observed data (Qian and Murphy, 2011). For a fixed value of *ω*, let ${\hat{Q}}_{\omega ,n}(x,a)=\omega {\hat{Q}}_{Y,n}(x,a)+(1-\omega ){\hat{Q}}_{Z,n}(x,a)$ and subsequently let ${\hat{d}}_{\omega ,n}(x)=\text{arg }{\text{max}}_{a\in A}{\hat{Q}}_{\omega ,n}(x,a)$ be the plug-in estimator of $d_{\omega }^{\text{opt}}(x)$. Given ${\hat{Q}}_{Y,n}$ and ${\hat{Q}}_{Z,n}$, ${\hat{d}}_{\omega ,n}(x)$ can be computed for each *ω* ∈ [0, 1].

The joint distribution of (***X***, *A, Y, Z*) is

$$f(X,A,Y,Z)=f(Y,Z|X,A)f(A|X)f(X)\;=f(Y,Z|X,A)f(X)\frac{\text{exp}\left[X^{⊤}\beta 1\left\{A=d_{\omega }^{\text{opt}}(X)\right\}\right]}{1+\text{exp}\left(X^{⊤}\beta \right)}.$$

Assuming that *f*(*Y, Z*|***X***, *A*) and *f*(***X***) do not depend on *ω* or *β*, the likelihood for (*ω, β*) is

(1) $$L_{n}(\omega ,\beta )\propto \prod_{i=1}^{n}\frac{\text{exp}\left[X_{i}^{⊤}\beta 1\left\{A_{i}=d_{\omega }^{\text{opt}}\left(X_{i}\right)\right\}\right]}{1+\text{exp}\left(X_{i}^{⊤}\beta \right)},$$

which depends on the unknown function $d_{\omega }^{\text{opt }}$. Plugging in ${\hat{d}}_{\omega ,n}$ for $d_{\omega }^{\text{opt }}$ into (7) yields the pseudo-likelihood

(2) $${\hat{L}}_{n}(\omega ,\beta )\propto \prod_{i=1}^{n}\frac{\text{exp}\left[X_{i}^{⊤}\beta 1\left\{A_{i}={\hat{d}}_{\omega ,n}\left(X_{i}\right)\right\}\right]}{1+\text{exp}\left(X_{i}^{⊤}\beta \right)}.$$

If we let ${\hat{\omega }}_{n}$ and ${\hat{\beta }}_{n}$ denote the maximum pseudo-likelihood estimators obtained by maximizing (2), then an estimator of the utility function is ${\hat{u}}_{n}(y,z)=u\left(y,z;{\hat{\omega }}_{n}\right)={\hat{\omega }}_{n}y+\left(1-{\hat{\omega }}_{n}\right)z$ and expit $\left(x^{⊤}{\hat{\beta }}_{n}\right)$ is an estimator of the probability that a patient presenting with covariates ***x*** would be treated optimally under standard care. An estimator of the optimal policy at ***x*** is ${\hat{d}}_{{\hat{\omega }}_{n},n}(x)=\text{arg }{\text{max}}_{a\in A}{\hat{Q}}_{{\hat{\omega }}_{n},n}(x,a)$.

Because the pseudo-likelihood given in (2) is non-smooth in *ω*, standard gradient-based optimization algorithms cannot be used. However, for a given *ω*, it is straightforward to compute the profile estimator ${\hat{\beta }}_{n}(\omega )=\text{arg }{\text{max}}_{\beta \in {\mathbb{R}}^{p}}{\hat{L}}_{n}(\omega ,\beta )$. We can compute the profile pseudo-likelihood estimator over a grid of values for *ω* and select the point on the grid yielding the largest pseudo-likelihood. The algorithm to construct (${\hat{\omega }}_{n}$, ${\hat{\beta }}_{n}$) is given in Algorithm 1 below. Step (3) can be accomplished using logistic regression. The theoretical properties of this estimator are discussed in Section 3.


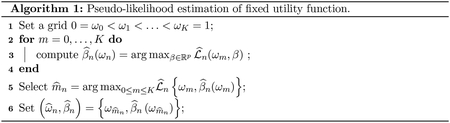


### 2.2 Patient-specific Utility

Outcome preferences can vary widely across patients in some application domains, including schizophrenia (Kinter, 2009; Strauss et al., 2010) and pain management (Gan et al., 2004). To accommodate this setting, we assume that the utility function takes the form *u*(*y, z*; ***x***, *ω*) = *ω*(***x***)*y* + {1 − *ω*(***x***)}*z* where ω:X→[0,1] is a smooth function. For illustration, we let *ω*(***x***; *θ*) = expit(***x***^⊤^*θ*) where *θ* is an unknown parameter. Misspecified utility models are discussed in the Appendix. Define *Q*_*θ*_(***x***, *a*) = *ω*(***x***; *θ*)*Q*_*Y*_ (***x***, *a*) + {1 − *ω*(***x***; *θ*)}*Q*_*Z*_(***x***, *a*) and define $d_{\theta }^{\text{opt}}(x)=\text{arg }{\text{max}}_{a\in A}Q_{\theta }(x,a)$. Let ${\hat{Q}}_{Y,n}$ and ${\hat{Q}}_{Z,n}$ denote estimators of *Q*_*Y*_ and *Q*_*Z*_ obtained from regression models fit to the observed data. For a fixed value of *θ*, let ${\hat{Q}}_{\theta ,n}(x,a)=\omega (x;\theta ){\hat{Q}}_{Y,n}(x,a)+\{1-\omega (x;\theta )\}{\hat{Q}}_{Z,n}(x,a)$ and subsequently let ${\hat{d}}_{\theta ,n}(x)=\text{arg }{\text{max}}_{a\in A}{\hat{Q}}_{\theta ,n}(x,a)$ be the plug-in estimator of $d_{\theta }^{\text{opt}}(x)$. Assume that decisions are made according to the model $\text{Pr}\left\{A=d_{\theta }^{\text{opt}}(x)|X=x\right\}=\text{expit}\left(x^{⊤}\beta \right)$. We compute the estimators (${\hat{\theta }}_{n}$, ${\hat{\beta }}_{n}$) of (*θ, β*) by maximizing the pseudo-likelihood

(3) $${\hat{L}}_{n}(\theta ,\beta )\propto \prod_{i=1}^{n}\frac{\text{exp}\left[X_{i}^{⊤}\beta 1\left\{A_{i}={\hat{d}}_{\theta ,n}\left(X_{i}\right)\right\}\right]}{1+\text{exp}\left(X_{i}^{⊤}\beta \right)}.$$

An estimator for the utility function is ${\hat{u}}_{n}(y,z;x)=\omega \left(x;{\hat{\theta }}_{n}\right)y+\left\{1-\omega \left(x;{\hat{\theta }}_{n}\right)\right\}z$ and an estimator for the optimal decision function is ${\hat{d}}_{{\hat{\theta }}_{n},n}$. The model, as stated is not identifiable. However, we show below that it is identifiable under the following conditions.

**Assumption 4** *The following conditions hold*.

1. $\beta \in B\subset {\mathbb{R}}^{p}$ *and* $\theta \in \Theta \subset {\mathbb{R}}^{q}$, *where* B *and* Θ *are compact*.
2. *β*_0_ ≠ 0.
3. X *is bounded* ($X\in X\subset {\mathbb{R}}^{p}$ a.s.).
4. *Let* $X_{S}$ *be the collection of subsets* of X *consisting of sets of the form* $\left\{x\in X:d_{\theta }\left(x\right)\neq d_{\theta_{0}}\left(x\right)\right\}$ *for θ* ∈ Θ \ {*θ*_0_}, *together with the complements of these sets. Then:*
  - *For all* $X_{S}\in X_{S}$, 0 < Pr(***X*** ∈ *X*_*S*_) *<* 1, *and*
  - *E* (***XX***^*T*^/***X*** ∈ *X*_*S*_) *is full rank* $\forall X_{S}\in X_{S}$.

**Theorem 5 (Identifiability)** *Under Assumption 4*, (*θ*_0_, *β*_0_) *is uniquely identified under the model given by $L_{n}(\theta ,\beta )$*.

**Remark 6** *A less technical but sufficient condition is to assume that* (*β*_0_, *θ*_0_) *satisfies* $\text{Pr}\left\{A=d_{\theta_{0}}\underline{(}X)|X\right\}>1/2$ *almost surely, i.e., that clinical decisions are always better than a coin toss. A proof of sufficiency is given in the* Appendix.

As before, the pseudo-likelihood given in (3) is non-smooth in *θ* and standard gradient-based optimization methods cannot be used. It is again straightforward to compute the profile pseudo-likelihood estimator ${\hat{\beta }}_{n}(\theta )=\text{arg }{\text{max}}_{\beta \in {\mathbb{R}}^{p}}{\hat{L}}_{n}(\theta ,\beta )$ for any $\theta \in {\mathbb{R}}^{p}$. However, because it is computationally infeasible to compute ${\hat{\beta }}_{n}(\theta )$ for all *θ* on a grid if *θ* is of moderate dimension, we generate a random walk through the parameter space using the Metropolis algorithm as implemented in the metrop function in the R package mcmc (Geyer and Johnson, 2017) and compute the profile pseudo-likelihood for each *θ* on the random walk. Let ${\tilde{L}}_{n}(\theta )={\text{max}}_{\beta \in {\mathbb{R}}^{p}}{\hat{L}}_{n}(\theta ,\beta )$. We can compute ${\tilde{L}}_{n}(\theta )={\hat{L}}_{n}\left\{\theta ,{\hat{\beta }}_{n}(\theta )\right\}$ by estimating ${\hat{\beta }}_{n}(\theta )$ using logistic regression as described in Section 2.1. The algorithm to construct a random walk through the parameter space is given in Algorithm 2 below. After generating a chain (*θ*^1^, *…, θ*^*B*^), we select the *θ*^*k*^ that leads to the largest value of ${\tilde{L}}_{n}\left(\theta^{k}\right)$ as the maximum pseudo-likelihood estimator. Standard practice is to choose the variance of the proposal distribution, *σ*^2^, so that the acceptance proportion is between 0.25 and 0.5 (Geyer and Johnson, 2017).


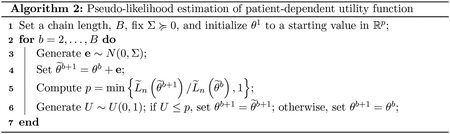


## 3. Theoretical Results

Here we state a number of theoretical results pertaining to the proposed pseudo-likelihood estimation method for utility functions. We state results for a patient-specific utility function; the setting where the utility function is fixed is a special case. All proofs are deferred to the Appendix.

We assume that $\text{Pr}\left\{A=d_{U}^{\text{opt}}(x)|X=x\right\}=\text{expit}\left(x^{⊤}\beta_{0}\right)$ and that the true utility function is *u*(*y, z*; ***x***, *θ*_0_) = *ω* (***X***; *θ*_0_)*y* + {1 − *ω* (***X***; *θ*_0_)}*z*, where *ω*(***X***; *θ*) has bounded continuous derivative on compact sets and $d_{\theta_{0}}^{\text{opt}}(X)=d_{\theta }^{\text{opt}}(X)$ almost surely implies *θ* = *θ*_0_, i.e., the model introduced in Section 2.2 is well-defined and correctly specified with true parameters $\beta_{0}\in {\mathbb{R}}^{p}$ and $\theta_{0}\in {\mathbb{R}}^{d}$. We further assume that the estimators ${\hat{Q}}_{Y,n}(x,a)$ and ${\hat{Q}}_{Z,n}(x,a)$ are pointwise consistent for all ordered pairs (***x***, *a*). Along with Assumptions 1–3, we implicitly assume that the densities *f*(*Y, Z*|***X***, *A*) and *f*(***X***) exist. The following result states the consistency of the maximum pseudo-likelihood estimators for the utility function and the probability of optimal treatment. The proof involves verifying the conditions of Theorem 2.12 of Kosorok (2008).

**Theorem 7 (Consistency with patient-specific utility)** *Let the maximum pseudo- likelihood estimators be as in* Section 2.2, $\left({\hat{\theta }}_{n},{\hat{\beta }}_{n}\right)=\text{arg }{\text{max}}_{\theta \in {\mathbb{R}}^{p},\beta \in B}{\hat{L}}_{n}(\theta ,\beta )$. *Assume that* B *is a compact set with* $\beta_{0}\in B$ and that ‖EX‖<∞ *Then*, $\left\|{\hat{\theta }}_{n}-\theta_{0}\right\|\overset{P}{\to }0$ *and* $\left\|{\hat{\beta }}_{n}-\beta_{0}\right\|\overset{P}{\to }0\;as\;n\to \infty$, *where* ∥ · ∥ *is the Euclidean norm*.

Let $V_{\theta }(d)=E\{u(Y,Z;X,\theta )|A=d(X)\}$ be the mean composite outcome in a population where decisions are made according to *d*. The following result establishes the consistency of the value of the estimated optimal policy. The proof uses general theory developed by Qian and Murphy (2011).

**Theorem 8 (Value consistency with patient-specific utility)** *Let ${\hat{\theta }}_{n}$ be the maximum pseudo-likelihood estimator for θ and let ${\hat{d}}_{{\hat{\theta }}_{n},n}$ be the associated estimated optimal policy. Then, under the given assumptions*, $\left|V_{\theta_{0}}\left({\hat{d}}_{{\hat{\theta }}_{n},n}\right)-V_{\theta_{0}}\left(d_{\theta_{0}}^{\text{opt}}\right)\right|\overset{P}{\to }0\;as\;n\to \infty$.

Next, we derive the convergence rate and asymptotic distribution of (${\hat{\theta }}_{n}$, ${\hat{\beta }}_{n}$). Assume that X is a bounded subset of ${\mathbb{R}}^{p}$ and let $\|\cdot {\|}_{X}$ be the sup norm over X, i.e., for f:X→ℝ, $\left\|f{\|}_{X}={\text{sup}}_{x\in X}|f(x)\right|$. Let ${\dot{\omega }}_{\theta }(x)=(\partial /\partial \theta )\omega (x;\theta )$. Assume that $\|\|{\dot{\omega }}_{\theta_{0}}(x)\|{\|}_{X}<\infty$ and that ${\text{lim}}_{\theta \to \theta_{0}}\|\|{\dot{\omega }}_{\theta }(x)-{\dot{\omega }}_{\theta_{0}}(x)\|{\|}_{X}=0$. Define *R*_*Y*_ (***x***) = *Q*_*Y*_ (***x***, 1) − *Q*_*Y*_ (***x***, −1) to be the treatment contrast for outcome *Y* at patient covariates ***X*** = ***x***; define *R*_*Z*_(***x***) = *Q*_*Z*_(***x***, 1) − *Q*_*Z*_(***x***, −1) analogously. Let *R*_0_(***x***) = *R*_*Y*_ (***x***) − *R*_*Z*_(***x***) denote the difference in the treatment contrasts across the two outcomes. Similarly, define ${\hat{R}}_{Y,n}(x)={\hat{Q}}_{Y,n}(x,1)-{\hat{Q}}_{Y,n}(x,-1)$, ${\hat{R}}_{Z,n}(x)={\hat{Q}}_{Z,n}(x,1)-{\hat{Q}}_{Z,n}(x,-1)$, and ${\hat{R}}_{0,n}(x)={\hat{R}}_{Y,n}(x)-{\hat{R}}_{Z,n}(x)$. Define *D*_*θ*_(***x***) = *ω*(***x***; *θ*)*R*_*Y*_ (***x***) + {1 − *ω*(***x***; *θ*)}*R*_*Z*_(***x***) to be the convex combination of treatment contrasts dictated by *ω*(***x***; *θ*) and let ${\hat{D}}_{\theta ,n}(x)=\omega (x;\theta ){\hat{R}}_{Y,n}(x)+\{1-\omega (x;\theta )\}{\hat{R}}_{Z,n}(x)$. Note that $d_{\theta }^{\text{opt}}(x)=\text{sign}\left\{D_{\theta }(x)\right\}$ and ${\hat{d}}_{\theta ,n}(x)=\text{sign}\left\{{\hat{D}}_{\theta ,n}(x)\right\}$. Further define

$$P_{\beta }(x)=\text{expit}\left(x^{⊤}\beta \right),$$

$$\psi_{i,A}=\left[1\left\{A_{i}=d_{\theta_{0}}^{\text{opt}}\left(X_{i}\right)\right\}-P_{\beta_{0}}\left(X_{i}\right)\right]X_{i},$$

$$I_{n}(\beta )=E_{n}\left[P_{\beta }(X)\left\{1-P_{\beta }(X)\right\}X\;X^{⊤}\right],$$

$$I_{0}=E\left[P_{\beta_{0}}(X)\left\{1-P_{\beta_{0}}(X)\right\}XX^{⊤}\right].$$

We use the following regularity conditions.

**Assumption 9** *There exist independent and identically distributed influence vectors $\psi_{1,Y},\psi_{2,Y},\ldots \in {\mathbb{R}}^{q_{1}}$, and $\psi_{1,Z},\psi_{2,Z},\ldots \in {\mathbb{R}}^{q_{2}}$, and vector basis functions ϕ*_*Y*_ (***x***) *and ϕ*_*Z*_(***x***) *such that both*

$${\left\|\sqrt{n}\left\{{\hat{R}}_{Y,n}(x)-R_{Y}(x)\right\}-\phi_{Y}{\left(x\right)}^{⊤}n^{-1/2}\sum_{i=1}^{n}\psi_{i,Y}\right\|}_{X}=o_{P}(1)$$

and

$${\left\|\sqrt{n}\left\{{\hat{R}}_{Z,n}(x)-R_{Z}(x)\right\}-\phi_{Z}{\left(x\right)}^{⊤}n^{-1/2}\sum_{i=1}^{n}\psi_{i,Z}\right\|}_{X}=o_{P}(1).$$

*Let $Z_{Y,n}=n^{-1/2}\sum_{i=1}^{n}\psi_{i,Y}$, $Z_{Z,n}=n^{-1/2}\sum_{i=1}^{n}\psi_{i,Z}$, $Z_{A,n}=n^{-1/2}\sum_{i=1}^{n}\psi_{i,A}$ and q* = *q*_1_ + *q*_2_. *Furthermore, assume that* ${\left\|R_{Y}(x)\right\|}_{X}$, ${\left\|R_{Z}(x)\right\|}_{X}$, $\|\|\phi_{Y}(x)\|{\|}_{X}$ *and* $\|\|\phi_{Z}(x)\|{\|}_{X}$ *are bounded by some M* < ∞. *Let* $\Sigma_{0}=E\left[{\left\{{\left(\psi_{1,Y}^{⊤},\psi_{1,Z}^{⊤},\psi_{1,A}^{⊤}\right)}^{⊤}\right\}}^{⊗2}\right]$ *be positive definite and finite, where u*^⊗2^ = *uu*^⊤^.

**Assumption 10** *The following conditions hold*.

1. *The random variable $D_{\theta_{0}}(X)$ has a continuous density function f in a neighborhood of 0 with f*_0_ = *f*(0) ∈ (0, ∞);
2. *The conditional distribution of* ***X*** *given that $\left|D_{\theta_{0}}(X)\right|\leq \epsilon$ converges to a non-degenerate distribution as ϵ* ↓ 0*;*
3. *There exist δ*_1_, *δ*_2_ *>* 0 *such that*
$$\underset{\epsilon ↓0}{\text{lim}}\underset{t\in S^{d}}{\text{inf}}\text{Pr}\left[\left|X^{⊤}\beta_{0}\right|\geq \delta_{1},\left|\left\{R_{Y}(X)-R_{Z}(X)\right\}{\dot{\omega }}_{\theta_{0}}{\left(X\right)}^{⊤}t\right|\geq \delta_{1}||D_{\theta_{0}}(X)|\leq \epsilon \right]\geq \delta_{2},$$
  *where S*^*d*^ *is the d-dimensional unit sphere*.

**Assumption 11** *Define, for* $Z_{Y}\in {\mathbb{R}}^{q_{1}}$, $Z_{Z}\in {\mathbb{R}}^{q_{2}}$, *and* $U\in {\mathbb{R}}^{d}$,

(4) $$\left(Z_{Y},Z_{Z},U\right)\mapsto k_{0}\left(Z_{Y},Z_{Z},U\right)=E[X\left\{2P_{\beta_{0}}(X)-1\right\}\cdot |\omega \left(X;\theta_{0}\right)R_{Y}(X)\phi_{Y}{\left(X\right)}^{⊤}Z_{Y}+\left\{1-\omega \left(X;\theta_{0}\right)\right\}R_{Z}(X)\phi_{Z}{\left(X\right)}^{⊤}Z_{Z}+R_{0}(X){\dot{\omega }}_{\theta_{0}}{\left(X\right)}^{⊤}U||D_{\theta_{0}}(X)=0].$$

*Assume that* $U\mapsto \beta_{0}^{⊤}k_{0}\left(Z_{Y},Z_{Z},U\right)$ *has a unique, finite minimum over* ${\mathbb{R}}^{d}$ *for all* ${\left(Z_{Y}^{⊤},Z_{Z}^{⊤}\right)}^{⊤}\in {\mathbb{R}}^{q}$.

**Remark 12** *Assumption 9 establishes a rate of convergence for the estimated Q-functions and is automatically satisfied if the Q-functions are estimated using linear or generalized linear models with or without interactions or higher order terms. Assumption 10 is needed to ensure that there is positive probability of patients with* ***x*** *values near the boundary between where each treatment is optimal. Assumption 11 is standard in M-estimation*.

Let (${\hat{\theta }}_{n}$, ${\hat{\beta }}_{n}$) be the maximum pseudo-likelihood estimators given in Section 2.2. The following theorem states the asymptotic distribution of (${\hat{\theta }}_{n}$, ${\hat{\beta }}_{n}$).

**Theorem 13 (Asymptotic distribution)** *Under the given regularity conditions*

(5) $$\sqrt{n}\left(\begin{array}{c} {\hat{\theta }}_{n}-\theta_{0}\\ {\hat{\beta }}_{n}-\beta_{0} \end{array}\right)⇝\left(\begin{array}{c} U\\ I_{0}^{-1}\left\{Z_{A}-k_{0}\left(Z_{Y},Z_{Z},U\right)\right\} \end{array}\right)\equiv \left(\begin{array}{l} U\\ B \end{array}\right),$$

*where* ${\left(Z_{Y}^{⊤},Z_{Z}^{⊤},Z_{A}^{⊤}\right)}^{⊤}\sim N\left(0,\sum_{0}\right)$, *and* $U=\text{arg }{\text{min}}_{u\in {\mathbb{R}}^{d}}\beta_{0}^{⊤}k_{0}\left(Z_{Y},Z_{Z},u\right)$.

Let $\overset{P}{\underset{Z*}{⇝}}$ denote convergence in probability over Z*, as defined in Section 2.2.3 and Chapter 10 of Kosorok (2008). Theorem 14 below establishes the validity of a parametric bootstrap procedure for approximating the sampling distribution of (${\hat{\theta }}_{n}$, ${\hat{\beta }}_{n}$).

**Theorem 14 (Parametric bootstrap)** *Assume ${\hat{\Sigma }}_{n}=\Sigma_{0}+o_{P}(1)$ and $h_{n}={\hat{v}}_{n}n^{-1/5}$, where ${\hat{v}}_{n}\overset{P}{\to }v_{0}\in (0,\infty )$ and v*_0_ is the standard error of $D_{\theta_{0}}(X)$. Assume the regularity conditions given above hold. Let $Z^{*}\sim N\left(0,I^{r\times r}\right)$, where I^r×r^ *is an r* × *r identity matrix and r* = *q* + p. Let ${\tilde{Z}}_{n}={\hat{\Sigma }}_{n}^{1/2}Z^{*}={\left({\tilde{Z}}_{Y}^{⊤},{\tilde{Z}}_{Z}^{⊤},{\tilde{Z}}_{A}^{⊤}\right)}^{⊤}$, where

$${\hat{\Sigma }}_{n}^{1/2}=\left[\begin{array}{cc} {\hat{\Sigma }}_{1}^{1/2} & 0\\ {\hat{\Sigma }}_{2}{\hat{\Sigma }}_{1}^{-1/2} & {\left({\hat{\Sigma }}_{2}-{\hat{\Sigma }}_{21}{\hat{\Sigma }}_{1}^{-1}{\hat{\Sigma }}_{12}\right)}^{1/2} \end{array}\right],$$

${\hat{\Sigma }}_{1}$ *is the top left q* × *q block of ${\hat{\Sigma }}_{n}$ (corresponding to Z*_*Y*_ *and Z*_*Z*_*), ${\hat{\Sigma }}_{2}$ is the lower right p* × *p block, ${\hat{\Sigma }}_{21}$. is the upper right q* × *p block, ${\hat{\Sigma }}_{12}={\hat{\Sigma }}_{21}^{⊤}$, and the matrix square roots are the symmetric square roots obtained from the associated Eigenvalue decompositions. Let*

$${\tilde{T}}_{n}\left(X,Z_{Y},Z_{Z}\right)=\omega \left(X;{\hat{\theta }}_{n}\right){\hat{R}}_{Y,n}(X)\phi_{Y}{\left(X\right)}^{⊤}Z_{Y}+\left\{1-\omega \left(X;{\hat{\theta }}_{n}\right)\right\}{\hat{R}}_{Z,n}(X)\phi_{Z}{\left(X\right)}^{⊤}Z_{Z}$$

*and define*

$${\tilde{k}}_{n}\left(Z_{Y},Z_{Z},U\right)=E_{n}[X\left\{2P_{{\hat{\beta }}_{n}}(X)-1\right\}\cdot |{\tilde{T}}_{n}\left(X,Z_{Y},Z_{Z}\right)+\left\{{\hat{R}}_{Y,n}(X)-{\hat{R}}_{Z,n}(X)\right\}{\dot{\omega }}_{{\hat{\theta }}_{n}}{\left(X\right)}^{⊤}U|\cdot h_{n}^{-1}\phi_{0}\left\{{\hat{D}}_{{\hat{\theta }}_{n},n}(X)/h_{n}\right\}]\times {\left\{E_{n}\left[h_{n}^{-1}\phi_{0}\left\{{\hat{D}}_{{\hat{\theta }}_{n},n}(X)/h_{n}\right\}\right]\right\}}^{-1},$$

*where ϕ*_0_ *is the standard normal density. Define* ${\tilde{U}}_{n}=\text{arg }{\text{min}}_{u\in {\mathbb{R}}^{d}}{\hat{\beta }}_{n}^{⊤}{\tilde{k}}_{n}\left({\tilde{Z}}_{Y},{\tilde{Z}}_{Z},u\right)$ *and* ${\tilde{B}}_{n}=I_{n}{\left({\hat{\beta }}_{n}\right)}^{-1}\left\{{\tilde{Z}}_{A}-{\tilde{k}}_{n}\left({\tilde{Z}}_{Y},{\tilde{Z}}_{Z},{\tilde{U}}_{n}\right)\right\}$. *Then*,

(6) $$\left(\begin{array}{c} {\tilde{U}}_{n}\\ {\tilde{B}}_{n} \end{array}\right)\overset{P}{\underset{{\tilde{Z}}^{*}}{⇝}}\left(\begin{array}{l} U\\ B \end{array}\right),$$

*where* (*U*^⊤^, *B*^⊤^)^⊤^ *is as defined in Theorem 13*.

If we fix a large number of bootstrap replications, *B*, then $\left({\tilde{U}}_{n,b},{\tilde{B}}_{n,b}\right),b=1,\ldots ,B$ will provide an approximation to the sampling distribution of the maximum pseudo-likelihood estimators. In Sections 4 and 5, we demonstrate the use of the bootstrap to test for heterogeneity of patient preferences.

**Remark 15** *In Theorem 13, it can be seen that when β*_0_ *only involves an intercept, there is no relationship between β*_0_ *and U, as the argmax of an objective function does not change under multiplication by a positive scalar. This relationship is more complex when β*_0_ *includes covariate effects. Theorem 13 also indicates that the asymptotic behaviors of* $\hat{\theta }$ *and* $\hat{\beta }$ *are driven largely by what happens at the boundary where* $D_{\theta_{0}}(X)=0$.

## 4. Simulation Experiments

### 4.1 Fixed Utility Simulations

To examine the finite sample performance of the proposed methods, we begin with the following simple generative model. Let ***X*** = (*X*_1_, *…, X*_5_)^⊤^ be a vector of independent normal random variables with mean 0 and standard deviation 0.5. Let treatment be assigned according to $\text{Pr}\left\{A=d_{\omega }^{\text{opt}}(x)|X=x\right\}=\rho$, i.e., the probability that the clinician correctly identifies the optimal treatment is constant across patients. Let *ϵ*_*Y*_ and *ϵ*_*Z*_ be independent normal random variables with mean 0 and standard deviation 0.5 and let *Y* = *A* (4*X*_1_ − 2*X*_2_ + 2) + *ϵ*_*Y*_ and *Z* = *A* (2*X*_1_ − 4*X*_2_ − 2) + *ϵ*_*Z*_. We estimated *Q*_*Y*_ and *Q*_*Z*_ using linear models, implemented the proposed method for a variety of *n*, *ω*, and *ρ* values, and examined ${\hat{\omega }}_{n}$, ${\hat{\rho }}_{n}$, and ${\hat{d}}_{{\hat{\omega }}_{n},n}$, across 500 Monte Carlo replications per scenario.

Table 1 contains mean estimates of *ω* and *ρ* across replications along with the associated standard deviation across replications, and estimated error rate defined as the proportion of subjects to whom the estimated optimal policy does not recommend the true optimal treatment; to better characterize sampling variability in the estimated error rate the last column displays the median along with the first and third quartiles of the sampling distribution of the estimated error rate.

The pseudo-likelihood method performs well at estimating both *ω* and *ρ*, with estimation improving with larger sample sizes as expected. Table 2 contains estimated values of the true optimal policy, a policy where the utility function is estimated (the proposed method), policies estimated to maximize the two outcomes individually (corresponding to fixing *ω* = 1 and *ω* = 0), and the standard of care. The value of the standard of care is the mean composite outcome under the generative model. For each policy, the value is estimated by generating a testing sample of size 500 with treatment assigned according to the policy and averaging utilities (calculated using the true *ω*) in the testing set. The standard deviation across replications is included in parentheses.

The column labeled “estimated *ω*” refers to the proposed method. We see that the proposed method produces values which increase with *n* and generally come close to the true optimal policy. In all settings, the proposed method offers significant improvement over the standard of care. The proposed method also offers improvement over policies to maximize each individual outcome.

To further examine the performance of the proposed method, we allow the probability of optimal treatment to depend on patient covariates. Let $\text{Pr}\left\{A=d_{\omega }^{\text{opt}}(X)\right\}=\text{expit}\left(0.5+X_{1}\right)$. This corresponds to the case where *β* = (0.5, 1, 0, *…*, 0)^⊤^, where the first element of *β* is an intercept. Let ***X***, *Y*, and *Z* be generated as described above. In this generative model, the probability that a patient is treated optimally in standard care is larger for patients with positive values of *X*_1_ and smaller for patients with negative values of *X*_1_. We applied the proposed method to 500 replications of this generative model for various *n* and *ω*. Table 3 contains mean estimates of *ω*, root mean squared error (RMSE) of ${\hat{\beta }}_{n}$, and the error rate along with its standard error and quartiles.

Estimation of the observational policy (as defined by *β*) improves with larger sample sizes. The probability that the estimated policy assigns the optimal treatment also increases with the sample size. The true value of *ω* does not affect estimation of *ω* or *β*.

Table 4 contains estimated values of the true optimal policy, a policy where the utility function is estimated (the proposed method), policies estimated to maximize each outcome individually, and the standard of care. Values are estimated from independent testing sets of size 500 as described above. The value under the standard of care is the mean composite outcome under the generative model.

The proposed method (found in the column labeled “estimated *ω*”) produces values that are close to the true optimal policy in large samples and a significant improvement over standard of care in small to moderate samples. We note that value under the standard of care differs across *ω*. When *ω* is close to 1, the composite outcome places more weight on *Y*, for which the magnitude of the association with *X*_1_ is larger. Because patients with larger values of *X*_1_ are more likely to be treated optimally in this generative model, the standard of care produces larger composite outcomes when *ω* is closer to 1. Likewise, the mean composite outcome under policies to maximize each individual outcome varies with the true value of *ω*.

### 4.2 Patient-specific Utility Simulations

Next, we examine the case where the utility function is allowed to vary across patients. Let ***X***, *Y*, and *Z* be generated as above. Again, assume that $\text{Pr}\left\{A=d_{\theta }^{\text{opt}}(X)\right\}=\text{expit}\left(0.5+X_{1}\right)$, i.e., *β* = (0.5, 1, 0, *…*, 0)^⊤^. Consider the composite outcome *U* = *ω*(***X***; *θ*)*Y* + {1 − *ω*(***X***, *θ*)}*Z*, where *ω*(***X***; *θ*) = expit(1 − 0.5*X*_1_), i.e., *θ* = (1, −0.5, 0, *…*, 0)^⊤^, where the first element of *θ* is an intercept. We implemented the proposed method for various *n* and examined estimation of *θ* and *β* across 500 replications. Each replication is based on a simulated Markov chain of length 10,000 as described in Section 2.2. Results are summarized in Table 5.

Larger sample sizes produce marginal decreases in the RMSE of ${\hat{\theta }}_{n}$. The estimated policy assigns the true optimal treatment more than 80% of the time for all sample sizes and the error rate decreases as the sample size increases. Table 6 contains estimated values of the true optimal policy, the policy estimated using the proposed method, policies estimated to maximize each outcome individually, and standard of care.

The proposed method produces policies that achieve significant improvement over the standard of care across sample sizes.

Finally, we examine the performance of the parametric bootstrap as described in Section 3. Let ***X*** be a bivariate vector of normal random variables with mean 0, standard deviation 0.5, and correlation zero. Let *Y* and *Z* be generated as above and let *β* = (2.5, 1, 0)^⊤^ where the first element of *β* is an intercept. Let *θ*_(1)_ be the vector *θ* with the first element removed. We are interested in testing the null hypothesis *H*_1_ : ∥*θ*_(1)_∥ = 0, which corresponds to a test for heterogeneity of patient preferences. The table below contains estimated power across 500 Monte Carlo replications under the null hypothesis, where the true value is *θ* = (1, 0, 0)^⊤^, and two alternative hypotheses: *H*_1_ : *θ* = (1, 4, 3)^⊤^, and *H*_2_ : *θ* = (1, 6, 6)^⊤^. All tests were conducted at level *α* = 0.05 and based on 1000 bootstrap samples. The last column in Table 7 shows the average agreement between the bootstrap and estimated optimal decision rule when *θ*_0_ = (1, 0, 0); the results suggest the decision rule is stable at sample sizes and generative models considered.

The proposed bootstrap procedure produces type I error rates near nominal levels under the null and moderate power in large samples under alternative hypotheses.

## 5. Case Study: The STEP-BD Standard Care Pathway

The Systematic Treatment Enhancement Program for Bipolar Disorder (STEP-BD) was a landmark study of the effects of antidepressants in patients with bipolar disorder (Sachs et al., 2007). In addition to a randomized trial assessing outcomes for patients given an antidepressant or placebo, the STEP-BD study also included a large-scale observational study, the standard care pathway. As our method requires observational data on clinical decision making, we apply the proposed method to the observational data from the STEP-BD standard care pathway to estimate decision rules for the use of antidepressants in patients with bipolar disorder. (Clearly, as clinicians are not generally assigning treatment according to their best clinical judgment in a randomized clinical trial, the proposed method is not applicable to the randomized pathway of STEP-BD.)

Although bipolar disorder is characterized by alternating episodes of depression and mania, recurrent depression is the leading cause of impairment among patients with bipolar disorder (Judd et al., 2002). However, the use of antidepressants has not become standard care in bipolar disorder due to the risk of antidepressants inducing manic episodes in certain patients (Ghaemi, 2008; Goldberg, 2008). Thus, the clinical decision in the treatment of bipolar disorder is whether to prescribe antidepressants to a specific patient in order to balance trade-offs between symptoms of depression, symptoms of mania, and other side effects of treatment.

**Figure 1:**
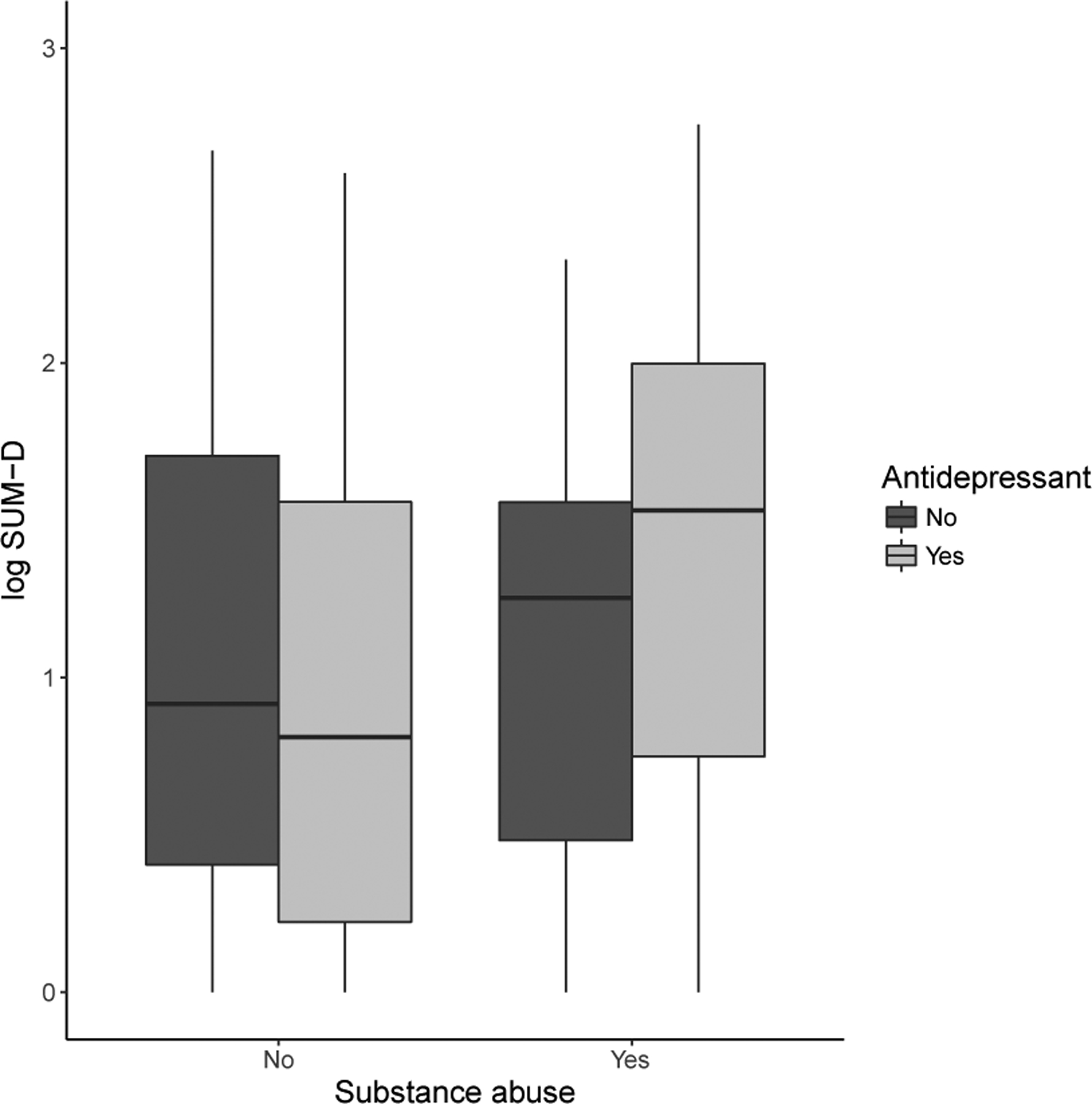
Box plots of log SUM-D by substance abuse and treatment.

**Figure 2:**
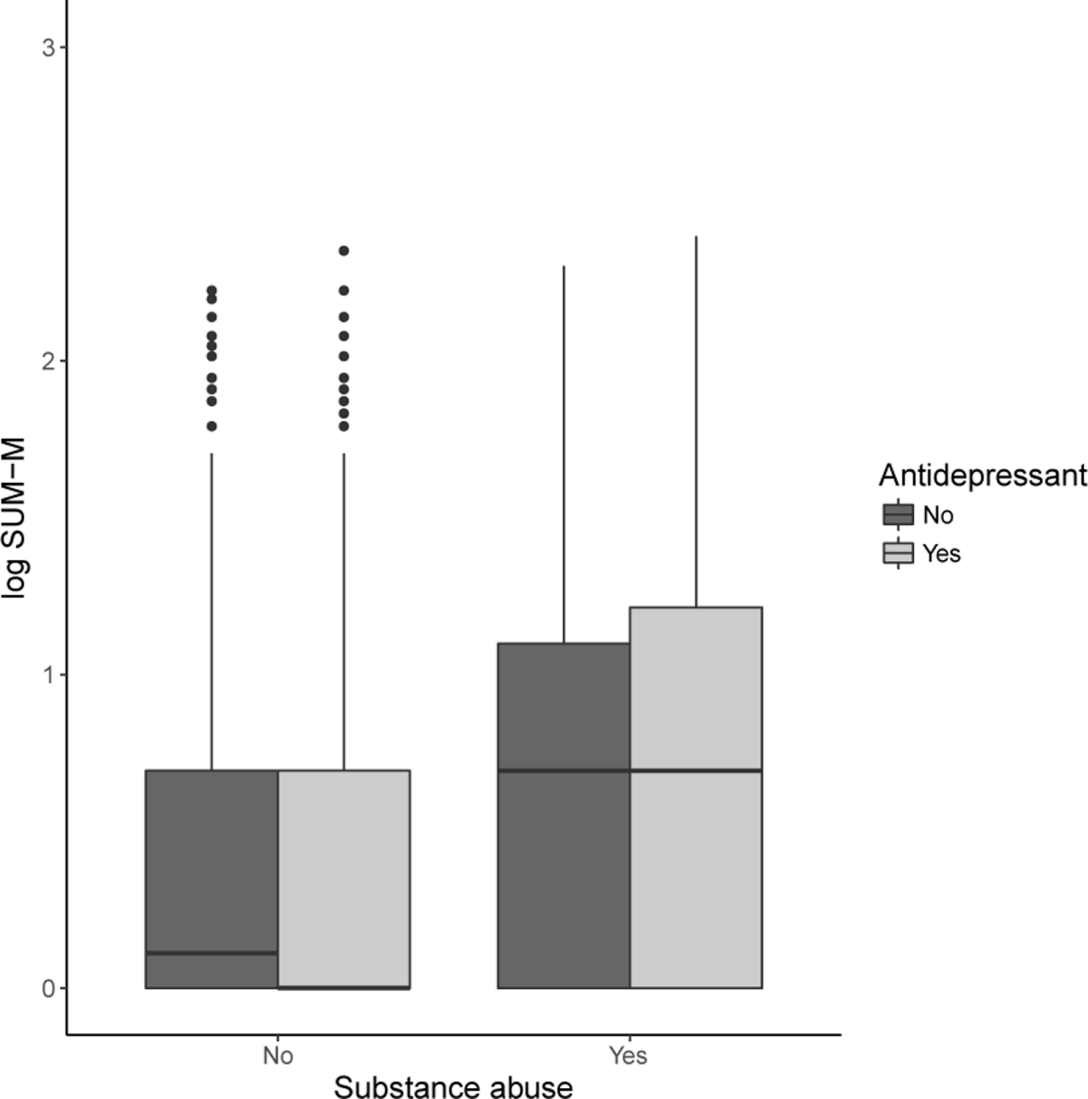
Box plots of log SUM-M by substance abuse and treatment.

We use the SUM-D score for depression symptoms and the SUM-M score for mania symptoms as outcomes. We consider a patient treated if they took any one of ten antidepressants that appear in the STEP-BD standard care pathway (Deseryl, Serzone, Citalopram, Escitalopram Oxalate, Prozac, Fluvoxamine, Paroxetine, Zoloft, Venlafaxine, or Bupropion). To generate candidate predictors for our model we made use of a complimentary randomized pathway in the STEP-BD trial. In this pathway, the patients are drawn from the same population, and the same variables are measured; however, treatment is randomly assigned so that there is no unmeasured confounding. Using step-wise variable selection to construct an outcome model from these data identified the following variables: mood elevation, anxiety, irritability, baseline SUM-M, and baseline SUM-D. We also used a step-wise logistic regression for the propensity score in the observational pathway to identify any additional potential confounders (Moodie et al., 2012). In addition to the variables in the outcome model, the logistic regression model identified race, insurance status, age, and substance abuse. The union of variables identified in through the randomized pathway and the propensity score were used in our models of the *Q*-functions and as tailoring variables in our treatment rules. Figure 1 contains box plots of SUM-D scores on the log scale by substance abuse and treatment. Figure 2 contains box plots of SUM-M scores on the log scale by substance abuse and treatment. For both outcomes, lower scores are more desirable. Figure 1 indicates that those without a history of substance abuse benefit from treatment with antidepressants. However, among those with a history of substance abuse, patients treated with antidepressants appear to have worse symptoms of depression. Figure 2 indicates that treatment has no effect on symptoms of mania among those without a history of substance abuse. However, among those with a history of substance abuse, it appears that treatment may be inducing manic episodes. Thus, a sensible treatment policy would be one that tends to prescribe antidepressants only to patients without a history of substance abuse.

We analyzed these data using the proposed method for optimizing composite outcomes. Results are summarized in Table 8 below. We estimated policies where both utility and probability of optimal treatment are fixed (fixed-fixed), where utility is fixed but probability of optimal treatment is assumed to vary between patients (fixed-variable), and where both utility and probability of optimal treatment are assumed to vary between patients (variable-variable). For both the fixed-variable policy and the variable-variable policy, we report $E_{n}\left\{\text{expit}\left(X^{⊤}{\hat{\beta }}_{n}\right)\right\}$ in place of ${\hat{\rho }}_{n}$ and for the variable-variable policy, we report $E_{n}\left\{\text{expit}\left(X^{⊤}{\hat{\theta }}_{n}\right)\right\}$ in place of ${\hat{\omega }}_{n}$. Thus, for parameters that are assumed to vary across patients, Table 8 contains the mean estimate in the sample. To evaluate each estimated policy, we used five-fold cross-validation of the inverse probability weighted estimator (IPWE) of the value for each outcome; i.e., for each fold, we used the training portion to estimate the optimal policy and propensity score, and we used the testing portion to compute the IPWE of the value; taking the average of the IPWE value estimates across folds yields the reported values. For both SUM-D and SUM-M, lower scores are preferred. Value is reported as the percent improvement over standard of care, calculated using the estimated utility function. Large percent improvements in value are preferred.

All estimated policies produce more desirable SUM-D scores and SUM-M scores compared to standard of care and improved value according to the estimated utility. Allowing the probability of optimal treatment to vary between patients leads to further improvements in value, as does allowing the utility function to vary between patients. All policies produce similar estimates for the probability of optimal treatment averaged across patients.

The resulting decision rules can be written as the sign of a linear combination of the covariates. As an example, the fixed-fixed policy assigns treatment with antidepressants when 0.032 − 0.001(age) − 0.646(substance abuse) − 0.007(mood elevation) + 0.007(medical insurance) + 0.129(white) is non-negative. The negative coefficient for substance abuse means that a history of substance abuse indicates that a patient should not be prescribed antidepressants. Prior research has shown that patients with a history of substance abuse are more likely to abuse antidepressants (Evans and Sullivan, 2014). This may contribute to the poor outcomes experienced by STEP-BD patients with a history of substance abuse who were treated with antidepressants. Table 9 displays estimates and standard errors of the components of ${\hat{\theta }}_{n}$ in the variable-variable policy. A test for preference heterogeneity based on 1000 bootstrap samples generated according to Theorem 14 yielded a p-value *<* 0.001.

As a secondary analysis, we use the SUM-D score and a side effect score as the outcomes. Eight side effects were recorded in the STEP-BD standard care pathway (tremors, dry mouth, sedation, constipation, diarrhea, headache, poor memory, sexual dysfunction, and increased appetite). Patients rated the severity of each side effect from 0 to 4 with larger values indicating more severe side effects. We took the mean score across side effects as the second outcome. Results are summarized in Table 10, reported analogously to those in Table 8.

Each estimated policy produces improved SUM-D scores and improved side effect scores compared to the standard of care. Each policy also produces improvement in value according to the estimated utility function. Again, allowing the utility function to vary between patients results in further improvements in value. Each policy produces similar estimates of the probability that patients are treated optimally in standard care. The variable-variable policy places more weight on SUM-D scores on average compared to the other policies. Table 11 displays estimates and standard errors of coefficients in ${\hat{\theta }}_{n}$ in the variable-variable policy. The bootstrap procedure for testing the null hypothesis that patient preferences are homogeneous based on 1000 bootstrap samples yielded a p-value *<* 0.001.

## 6. Discussion

The estimation of individualized treatment rules has been well-studied in the statistical literature. Existing methods have typically defined the optimal treatment rule as optimizing the mean of a fixed scalar outcome. However, clinical practice often requires consideration of multiple outcomes. Thus, there is a disconnect between existing statistical methods and current clinical practice. It is reasonable to assume that clinicians make treatment decisions for each patient with the goal of maximizing that patient’s utility. Therefore, it is natural to use observational data to estimate patient utilities from observed clinician decisions. This represents a new paradigm for the use of observational data in the context of precision medicine in that clinical decisions are viewed as a (noisy) surrogate for patient preferences and leveraged to improve the quality of a learned treatment rule and to generate new insights into heterogeneity in patient preferences.

The proposed methodology offers many opportunities for future research. In the present manuscript, we have considered only the simplest case— that of one decision time, two outcomes, and two possible treatments. Scenarios with more than two outcomes are discussed in the Appendix, and the simulation results there demonstrate that the proposed method performs well with three outcomes. Extensions to more than two treatments or multiple time points represent potential areas for future research. The proposed method requires positing a parametric model for the utility function. Model misspecification is discussed in the Appendix, and the simulation results there demonstrate that the proposed method performs reasonably well when important covariates are omitted from the model for the utility function. However, the use of semi- or non-parametric models is an important extension. A more technical direction for future work is a more nuanced study of the affect of boundary conditions on the resulting rate of convergence (see Assumption 10). Finally, while we have proposed our utility function estimator inside the framework of one-stage Q-learning, the pseudo-likelihood utility function estimator could be used alongside other existing one-stage optimal treatment policy estimators based on (augmented) inverse probability weighting (e.g., Zhao et al., 2012; Zhang et al., 2012a). There is a great future for the development of methods for optimizing composite outcomes in precision medicine and application of these methods in clinical studies.

## Figures and Tables

**Table 1:** Estimation results for simulations where utility and probability of optimal treatment are fixed.

*n*	*ω*	*ρ*	${\hat{\omega }}_{n}$	${\hat{\rho }}_{n}$	Error rate	Median(25th, 75th)
100	0.25	0.60	0.34 (0.24)	0.61 (0.08)	0.12 (0.13)	0.07 (0.03, 0.13)
			0.25 (0.05)	0.80 (0.04)	0.03 (0.02)	0.02 (0.01, 0.03)
	0.75	0.60	0.66 (0.24)	0.61 (0.07)	0.12 (0.13)	0.07 (0.03, 0.14)
			0.75 (0.05)	0.80 (0.04)	0.03 (0.02)	0.02 (0.01, 0.03)
200	0.25	0.60	0.28 (0.16)	0.61 (0.04)	0.07 (0.08)	0.04 (0.02, 0.10)
			0.25 (0.02)	0.80 (0.03)	0.01 (0.01)	0.01 (0.01, 0.02)
	0.75	0.60	0.72 (0.16)	0.61 (0.04)	0.07 (0.09)	0.03 (0.01, 0.08)
			0.75 (0.03)	0.80 (0.03)	0.01 (0.01)	0.01 (0.01, 0.02)
300	0.25	0.60	0.26 (0.11)	0.61 (0.03)	0.05 (0.06)	0.03 (0.01, 0.06)
			0.25 (0.02)	0.80 (0.02)	0.01 (0.01)	0.01 (0.00, 0.01)
	0.75	0.60	0.74 (0.13)	0.61 (0.03)	0.06 (0.07)	0.03 (0.01, 0.08)
			0.75 (0.02)	0.80 (0.02)	0.01 (0.01)	0.01 (0.01, 0.01)
500	0.25	0.60	0.25 (0.08)	0.61 (0.02)	0.04 (0.04)	0.02 (0.01, 0.04)
			0.25 (0.01)	0.80 (0.02)	0.01 (0.01)	0.01 (0.00, 0.01)
	0.75	0.60	0.75 (0.08)	0.61 (0.02)	0.04 (0.04)	0.02 (0.01, 0.05)
			0.75 (0.01)	0.80 (0.02)	0.01 (0.01)	0.01 (0.00, 0.01)

**Table 2:** Value results for simulations where utility and probability of optimal treatment are fixed.

*n*	*ω*	*ρ*	Optimal	Estimated *ω*	*Y* only	*Z* only	Standard of care
100	0.25	0.60	1.90 (0.07)	1.70 (0.38)	0.38 (0.11)	1.76 (0.08)	0.37 (0.24)
			1.90 (0.07)	1.89 (0.07)	0.39 (0.12)	1.76 (0.08)	1.14 (0.21)
	0.75	0.60	1.90 (0.06)	1.71 (0.37)	1.76 (0.08)	0.39 (0.12)	0.37 (0.23)
			1.90 (0.06)	1.89 (0.07)	1.76 (0.08)	0.39 (0.12)	1.14 (0.21)
200	0.25	0.60	1.90 (0.07)	1.82 (0.21)	0.39 (0.11)	1.76 (0.07)	0.39 (0.16)
			1.90 (0.07)	1.90 (0.06)	0.39 (0.11)	1.76 (0.07)	1.14 (0.14)
	0.75	0.60	1.90 (0.07)	1.82 (0.22)	1.76 (0.07)	0.38 (0.11)	0.39 (0.17)
			1.90 (0.07)	1.90 (0.06)	1.76 (0.07)	0.38 (0.11)	1.14 (0.15)
300	0.25	0.60	1.90 (0.07)	1.86 (0.13)	0.39 (0.11)	1.76 (0.07)	0.38 (0.14)
			1.90 (0.07)	1.89 (0.06)	0.39 (0.11)	1.76 (0.07)	1.14 (0.12)
	0.75	0.60	1.90 (0.06)	1.85 (0.17)	1.77 (0.07)	0.38 (0.11)	0.38 (0.14)
			1.90 (0.06)	1.90 (0.07)	1.77 (0.07)	0.38 (0.11)	1.13 (0.12)
500	0.25	0.60	1.90 (0.06)	1.88 (0.10)	0.39 (0.10)	1.76 (0.07)	0.38 (0.10)
			1.90 (0.06)	1.90 (0.07)	0.39 (0.11)	1.76 (0.07)	1.14 (0.09)
	0.75	0.60	1.89 (0.07)	1.88 (0.08)	1.77 (0.07)	0.39 (0.11)	0.38 (0.11)
			1.89 (0.07)	1.90 (0.07)	1.77 (0.07)	0.39 (0.11)	1.14 (0.09)

**Table 3:** Estimation results for simulations where utility is fixed and probability of optimal treatment is variable.

*n*	*ω*	${\hat{\omega }}_{n}$	RMSE of ${\hat{\beta }}_{n}$	Error rate	Median(25th, 75th)
100	0.25	0.34 (0.23)	1.32 (0.50)	0.10 (0.14)	0.04 (0.02, 0.10)
	0.75	0.71 (0.21)	1.37 (0.48)	0.10 (0.11)	0.06 (0.03, 0.12)
200	0.25	0.27 (0.13)	0.81 (0.30)	0.04 (0.08)	0.02 (0.01, 0.03)
	0.75	0.75 (0.15)	0.85 (0.29)	0.07 (0.07)	0.04 (0.02, 0.10)
300	0.25	0.26 (0.09)	0.60 (0.21)	0.03 (0.05)	0.01 (0.01, 0.02)
	0.75	0.75 (0.10)	0.63 (0.22)	0.04 (0.05)	0.03 (0.01, 0.07)
500	0.25	0.25 (0.03)	0.44 (0.14)	0.01 (0.01)	0.01 (0.00, 0.01)
	0.75	0.76 (0.07)	0.46 (0.16)	0.03 (0.04)	0.02 (0.01, 0.04)

**Table 4:** Value results for simulations where utility is fixed and probability of optimal treatment is variable.

*n*	*ω*	Optimal	Estimated *ω*	*Y* only	*Z* only	Standard of care
100	0.25	1.90 (0.07)	1.72 (0.40)	0.39 (0.12)	1.76 (0.07)	0.33 (0.23)
	0.75	1.90 (0.06)	1.75 (0.30)	1.76 (0.08)	0.39 (0.12)	0.56 (0.23)
200	0.25	1.90 (0.06)	1.85 (0.23)	0.37 (0.11)	1.76 (0.07)	0.34 (0.16)
	0.75	1.89 (0.06)	1.83 (0.18)	1.76 (0.07)	0.38 (0.11)	0.58 (0.16)
300	0.25	1.90 (0.06)	1.88 (0.16)	0.39 (0.10)	1.77 (0.07)	0.33 (0.14)
	0.75	1.90 (0.06)	1.87 (0.08)	1.76 (0.07)	0.40 (0.11)	0.57 (0.13)
500	0.25	1.90 (0.07)	1.89 (0.06)	0.39 (0.10)	1.76 (0.07)	0.33 (0.11)
	0.75	1.90 (0.07)	1.88 (0.07)	1.77 (0.07)	0.39 (0.11)	0.58 (0.10)

**Table 5:** Estimation results for simulations where both utility and probability of optimal treatment are variable.

*n*	RMSE of ${\hat{\theta }}_{n}$	RMSE of ${\hat{\beta }}_{n}$	Error rate	Median(25th, 75th)
100	0.85 (0.45)	1.28 (0.46)	0.10 (0.08)	0.06 (0.04, 0.13)
200	0.72 (0.31)	0.81 (0.27)	0.07 (0.06)	0.05 (0.04, 0.08)
300	0.63 (0.16)	0.63 (0.21)	0.05 (0.04)	0.04 (0.03, 0.06)
500	0.60 (0.09)	0.46 (0.15)	0.05 (0.02)	0.04 (0.03, 0.05)

**Table 6:** Value results for simulations where both utility and probability of optimal treatment are variable.

*n*	Optimal	Estimated *ω*	*Y* only	*Z* only	Standard of care
100	1.74 (0.06)	1.65 (0.14)	1.66 (0.06)	1.41 (0.08)	0.50 (0.21)
200	1.74 (0.06)	1.69 (0.11)	1.67 (0.06)	1.41 (0.08)	0.49 (0.15)
300	1.74 (0.06)	1.71 (0.07)	1.66 (0.06)	1.41 (0.07)	0.50 (0.13)
500	1.74 (0.06)	1.71 (0.07)	1.66 (0.06)	1.41 (0.08)	0.50 (0.11)

**Table 7:** Power of bootstrap test for homogeneity of utility function

*n*	Type 1 error	Power against *H*_1_	Power against *H*_2_	Stability
100	0.002	0.238	0.264	0.805
200	0.004	0.790	0.782	0.831
300	0.004	0.958	0.948	0.833
500	0.002	0.998	0.990	0.829

**Table 8:** Results of analysis of STEP-BD data for SUM-D and SUM-M.

Policy	SUM-D	SUM-M	Value (% improvement)	${\hat{\omega }}_{n}$	${\hat{\rho }}_{n}$
fixed-fixed	2.336	0.857	1.8%	0.039	0.431
fixed-variable	2.324	0.838	3.9%	0.039	0.440
variable-variable	2.321	0.804	8.3%	0.334	0.448
standard of care	2.480	0.868	0.0%	·	·

**Table 9:** Estimates of ${\hat{\theta }}_{n}$ in the variable-variable policy

	Intercept	Age	Substance	Mood elevation	Insurance	Race
Estimate	2.427	−.177	−1.666	−2.632	4.263	1.078
Standard error	3.039	0.953	2.746	2.238	3.241	3.274

**Table 10:** Results of analysis of STEP-BD data for SUM-D and Side effect score.

Policy	SUM-D	Side effect score	Value (% improvement)	${\hat{\omega }}_{n}$	${\hat{\rho }}_{n}$
fixed-fixed	2.377	0.156	5.2%	0.601	0.462
fixed-variable	2.384	0.159	5.7%	0.100	0.472
variable-variable	2.430	0.161	6.1%	0.378	0.487
standard of care	2.480	0.172	0.0%	·	·

**Table 11:** Estimates of ${\hat{\theta }}_{n}$ in the variable-variable policy

	Intercept	Age	Substance	Mood	Irritable	Anxiety	Insurance	Race
Estimate	−3.125	−4.614	2.094	−0.609	2.594	0.332	−3.493	−2.563
Standard error	2.257	2.300	2.395	2.603	2.610	2.538	2.599	2.449

## Footnotes

1. Nevertheless, one could imagine how these flexible estimators could integrated into our framework to reduce dependence on parametric models. See concluding remarks for additional discussion.

2. We gratefully acknowledge an anonymous referee for identifying this connection.

## Appendix A: Proofs

**Proof** [Proof of Theorem 5] Consider (β,θ)∈B×Θ and suppose

(7)
$$P_{\beta }{\left(X\right)}^{1\left\{Y=d_{\theta }(X)\right\}}(1-P_{\beta }{\left(X\right)}^{1-1\left\{Y=d_{\theta }(X)\right\}}=P_{\beta_{0}}{\left(X\right)}^{1\left\{Y=d_{\theta_{0}}(X)\right\}}(1-P_{\beta_{0}}{\left(X\right)}^{1-1\left\{Y=d_{\theta_{0}}(X)\right\}}\forall (X,Y)\text{ a}.\text{s}.$$

Let $X_{S}=\left\{X:d_{\theta }(X)=d_{\theta_{0}}(X)\right\}$. For all ***X*** ∈ *X*_*S*_, we have that $P_{\beta }(X)=P_{\beta_{0}}(X)\Rightarrow \beta =\beta_{0}$ by *4* in Assumption 4 which implies identifiability of *P*_*β*_(***X***) on *X*_*S*_.

Now suppose *θ* ≠ *θ*_0_, then since *β* = *β*_0_, we have by (7), that $\forall X\in X_{S}^{c}$, $P_{\beta }(X)=1-P_{\beta_{0}}(X)$ which by applying *4* in Assumption 4 again, we obtain that *β* = −*β*_0_. However, this is impossible by *2* in Assumption 4. Thus *θ* = *θ*_0_, and we obtain that (*β, θ*) = (*β*_0_, *θ*_0_). ■

**Proof** [Proof of Theorem 7] The log of the pseudo-likelihood is given by

$${\hat{l}}_{n}(\theta ,\beta )=E_{n}[X^{⊤}\beta 1\left\{A={\hat{d}}_{\theta ,n}(X)\right\}-\text{log}\left\{1+\text{exp}\left(X^{⊤}\beta \right)\right\}].$$

Let $\hat{m}(\cdot ,\cdot ;\theta ,\beta ):X\times A\to \mathbb{R}$ be defined by $\hat{m}(X,A;\theta ,\beta )=X^{⊤}\beta 1\left\{A={\hat{d}}_{\theta ,n}(X)\right\}-\text{log}\left\{1+\text{exp}\left(X^{⊤}\beta \right)\right\}$ and consider the class of functions $\left\{\hat{m}(\cdot ,\cdot ;\theta ,\beta ):\theta \in {\mathbb{R}}^{p},\beta \in B\right\}$. The class $\left\{\text{log}\left\{1+\text{exp}\left(X^{⊤}\beta \right)\right\}:\beta \in B\right\}$ is contained in a VC class by Lemma 9.9 (viii) and (v) of Kosorok (2008). By Theorem 9.3 of Kosorok (2008), this is also a Glivenko–Cantelli (GC) class.

Let $u(X,A;\theta )=\omega (X;\theta )\left\{{\hat{Q}}_{Y,n}(X,A)-{\hat{Q}}_{Z,n}(X,A)\right\}+{\hat{Q}}_{Z,n}(X,A)$, which lies in a VC class indexed by $\theta \in {\mathbb{R}}^{p}$ by Lemma 9.6 and Lemma 9.9 (viii), (vi), and (v) of Kosorok (2008). We have that

$$1\left\{A={\hat{d}}_{\theta ,n}\left(X\right)\right\}=1\left(A=1\right)1\left\{u\left(X,1;\theta \right)-u\left(X,-1,\theta \right)\geq 0\right\}+1(A=-1)1\{u(X,1;\theta )-u(X,-1,\theta )<0\}$$

and it follows that $1\left\{A={\hat{d}}_{\theta ,n}(X)\right\}$ is contained in a GC class indexed by $\theta \in {\mathbb{R}}^{p}$. From Corollary 9.27 (ii) of Kosorok (2008) it follows that $X^{⊤}\beta 1\left\{A={\hat{d}}_{\theta ,n}(X)\right\}$ lies in a GC class indexed by $(\theta ,\beta )\in {\mathbb{R}}^{p}\times B$ as long as ***X***^⊤^*β* is uniformly bounded by a function with finite mean, which holds as long as B is compact and ‖EX‖<∞. It follows that

$$\underset{(\theta ,\beta )\in {\mathbb{R}}^{p}\times B}{\text{sup}}\left|\left(E_{n}-E\right)\left[X^{⊤}\beta 1\left\{A={\hat{d}}_{\theta ,n}(X)\right\}-\text{log}\left\{1+\text{exp}\left(X^{⊤}\beta \right)\right\}\right]\right|\overset{P}{\to }0.$$

Next, define

$$\hat{M}(\theta ,\beta )=E\{\hat{m}(X,A;\theta ,\beta )\}=E\left(X^{⊤}\beta E\left[1\left\{A={\hat{d}}_{\theta ,n}(X)\right\}|X\right]\right)-E\text{log}\left\{1+\text{exp}\left(X^{⊤}\beta \right)\right\}$$

and note that $\hat{M}(\theta ,\beta )$ is continuous in *β*. The inside expectation of the first piece is

$$E\left[1\left\{A={\hat{d}}_{\theta ,n}(X)\right\}|X\right]=\text{expit}\left(X^{⊤}\beta_{0}\right)1\left\{{\hat{d}}_{\theta ,n}(X)=d_{\theta_{0}}^{\text{opt}}(X)\right\}+\left\{1-\text{expit}\left(X^{⊤}\beta_{0}\right)\right\}1\left\{{\hat{d}}_{\theta ,n}(X)\neq d_{\theta_{0}}^{\text{opt}}(X)\right\},$$

using the fact that $\text{Pr}\left\{A=d_{\theta_{0}}^{\text{opt}}(X)\right\}=\text{expit}\left(X^{⊤}\beta_{0}\right)$. Define *a*(***X***) = *Q*_*Y*_ (***X***, 1)−*Q*_*Y*_ (***X***, −1)−*Q*_*Z*_(***X***, 1) + *Q*_*Z*_(***X***, −1) and *b*(***X***) = *Q*_*Z*_(***X***, 1) − *Q*_*Z*_(***X***, −1). Similarly, define $\hat{a}(X)={\hat{Q}}_{Y,n}(X,1)-{\hat{Q}}_{Y,n}(X,-1)-{\hat{Q}}_{Z,n}(X,1)+{\hat{Q}}_{Z,n}(X,-1)$ and $\hat{b}(X)={\hat{Q}}_{Z,n}(X,1)-{\hat{Q}}_{Z,n}(X,-1)$. Then,

$$1\left\{{\hat{d}}_{\theta ,n}(X)=d_{\theta_{0}}^{\text{opt}}(X)\right\}=1[\{\omega (X;\theta )\hat{a}(X)+\hat{b}(X)\}\{\omega (X;\theta )a(X)+b(X)\}\geq 0]\;=1[\omega (X;\theta )\{\omega (X;\theta )a(X)\hat{a}(X)+\hat{a}(X)b(X)\}+\omega (X;\theta )a(X)\hat{b}(X)+\hat{b}(X)b(X)\geq 0],$$

and thus $E\left[1\left\{A={\hat{d}}_{\theta ,n}(X)\right\}|X\right]$ is continuous in *θ*.

Let $m(X,A;\theta ,\beta )=X^{⊤}\beta 1\left\{A=d_{\theta }^{\text{opt}}(X)\right\}-\text{log}\left\{1+\text{exp}\left(X^{⊤}\beta \right)\right\}$. Because the model is identifiable and $L_{n}(\theta ,\beta )$ is a parametric log-likelihood, Em(X,A;θ,β) has unique maximizers at *θ*_0_ and *β*_0_. Let ${\tilde{\theta }}_{n}$ and ${\tilde{\beta }}_{n}$ be the maximizers of $E\hat{m}(X,A;\theta ,\beta )$. Because $E\left\{\left|{\hat{d}}_{\theta ,n}(X)-d_{\theta }^{\text{opt}}(X)\right|\right\}\to 0$ in probability, for any $\theta \in {\mathbb{R}}^{d}$, $E\left[1\left\{A={\hat{d}}_{\theta ,n}(X)\right\}|X\right]-E\left[1\left\{A=d_{\theta }^{\text{opt}}(X)\right\}|X\right]\to 0$ in probability, uniformly in *θ* over compact subsets of ${\mathbb{R}}^{d}$, which implies that both ${\tilde{\theta }}_{n}\to \theta_{0}$ and $\tilde{\beta }\to \beta_{0}$ in probability. The claim now follows from Lemma 14.3 and Theorem 2.12 of Kosorok (2008).■

**Proof** [Proof of Theorem 8] Define $Q_{\theta_{0}}(x,a)$ and $Q_{{\hat{\theta }}_{n}}(x,a)$ as defined in Section 2. Let *u*(*Y*, *Z*; *A*, ***X***, *θ*) = *ω*(***X***; *θ*)*Q*_*Y*_ (***X***, *A*) + {1 − *ω*(***X***; *θ*)}*Q*_*Z*_(***X***, *A*). Under the given assumptions, for some constant 0 *< c <* ∞,

(8)
$$\left|V\left({\hat{d}}_{{\hat{\theta }}_{n},n}\right)-V\left(d_{\theta_{0}}^{\text{opt}}\right)\right|\leq c{\left|E{\left\{u\left(Y,Z;A,X,\theta_{0}\right)-{\hat{Q}}_{{\hat{\theta }}_{n},n}(X,A)\right\}}^{2}-E{\left\{u\left(Y,Z;A,X,\theta_{0}\right)-Q_{\theta_{0}}(X,A)\right\}}^{2}\right|}^{1/2}$$

by equation (3.1) of Qian and Murphy (2011) (see also Murphy, 2005). The right hand side of (8) converges in probability to 0 by the consistency of ${\hat{\theta }}_{n}$, consistency of ${\hat{Q}}_{Y,n}$ and ${\hat{Q}}_{Z,n}$, and the continuous mapping theorem. The result follows.■

**Proof** [Proof of Theorem 13] By definition of (${\hat{\theta }}_{n}$, ${\hat{\beta }}_{n}$),

$$0\leq {\hat{l}}_{n}\left({\hat{\theta }}_{n},{\hat{\beta }}_{n}\right)-{\hat{l}}_{n}\left(\theta_{0},\beta_{0}\right)\;=\sum_{i=1}^{n}[X_{i}^{⊤}{\hat{\beta }}_{n}1\left\{A_{i}={\hat{d}}_{{\hat{\theta }}_{n},n}\left(X_{i}\right)\right\}-X_{i}^{⊤}\beta_{0}1\left\{A_{i}={\hat{d}}_{\theta_{0},n}\left(X_{i}\right)\right\}-{\left({\hat{\beta }}_{n}-\beta_{0}\right)}^{⊤}X_{i}P_{\beta_{0}}\left(X_{i}\right)]-\frac{1}{2}\sqrt{n}{\left({\hat{\beta }}_{n}-\beta_{0}\right)}^{⊤}I_{n}\left(\beta_{*}\right)\sqrt{n}\left({\hat{\beta }}_{n}-\beta_{0}\right)\;=\sqrt{n}{\left({\hat{\beta }}_{n}-\beta_{0}\right)}^{⊤}n^{-1/2}\sum_{i=1}^{n}X_{i}\left[1\left\{A_{i}={\hat{d}}_{{\hat{\theta }}_{n},n}\left(X_{i}\right)\right\}-P_{\beta_{0}}\left(X_{i}\right)\right]-\frac{1}{2}\sqrt{n}{\left({\hat{\beta }}_{n}-\beta_{0}\right)}^{⊤}I_{n}\left(\beta_{*}\right)\sqrt{n}\left({\hat{\beta }}_{n}-\beta_{0}\right)+\sum_{i=1}^{n}X_{i}^{⊤}\beta_{0}\left[1\left\{A_{i}={\hat{d}}_{{\hat{\theta }}_{n},n}\left(X_{i}\right)\right\}-1\left\{A_{i}={\hat{d}}_{\theta_{0},n}\left(X_{i}\right)\right\}\right],$$

where *β*_*_ is a point between ${\hat{\beta }}_{n}$ and *β*_0_. Using the definition of a maximizer and letting ${\hat{u}}_{n}(\theta )=n^{-1/2}\sum_{i=1}^{n}X_{i}\left[1\left\{A_{i}={\hat{d}}_{\theta ,n}\left(X_{i}\right)\right\}-P_{\beta_{0}}\left(X_{i}\right)\right]$, we have that $\sqrt{n}\left({\hat{\beta }}_{n}-\beta_{0}\right)=I_{n}{\left(\beta_{*}\right)}^{-1}{\hat{u}}_{n}\left({\hat{\theta }}_{n}\right)$ by setting ${\frac{\partial }{\partial \beta }{\hat{l}}_{n}(\hat{\theta },\beta )|}_{\hat{\beta }}=0$, since $I_{n}\left(\beta_{*}\right)\overset{P}{\to }I_{0}$ and *I*_0_ is positive definite. Next, note that

$${\hat{u}}_{n}\left({\hat{\theta }}_{n}\right)=n^{-1/2}\sum_{i=1}^{n}X_{i}\left[1\left\{A_{i}={\hat{d}}_{{\hat{\theta }}_{n},n}\left(X_{i}\right)\right\}-1\left\{A_{i}=d_{\theta_{0}}^{\text{opt}}\left(X_{i}\right)\right\}\right]+Z_{A,n}\;=G_{n}\left(X\left[1\left\{A={\hat{d}}_{{\hat{\theta }}_{n},n}(X)\right\}-1\left\{A=d_{\theta_{0}}^{\text{opt}}(X)\right\}\right]\right)+Z_{A,n}+\sqrt{n}E\left(X\left[1\left\{A={\hat{d}}_{{\hat{\theta }}_{n},n}(X)\right\}-1\left\{A=d_{\theta_{0}}^{\text{opt}}(X)\right\}\right]\right)\;=Z_{A,n}+\sqrt{n}E\left(X\left[1\left\{A={\hat{d}}_{{\hat{\theta }}_{n},n}(X)\right\}-1\left\{A=d_{\theta_{0}}^{\text{opt}}(X)\right\}\right]\right)\left\{1+o_{P}(1)\right\}+o_{P}(1),$$

where $G_{n}f=n^{1/2}\left(E_{n}-E\right)f(X)$ and $Z_{A,n}=n^{-1/2}\sum_{i=1}^{n}\left[1\left\{A_{i}=d_{\theta_{0}}^{\text{opt}}\left(X_{i}\right)\right\}-P_{\beta_{0}}\left(X_{i}\right)\right]$. We also have $1\left\{A={\hat{d}}_{{\hat{\theta }}_{n},n}(X)\right\}-1\left\{A=d_{\theta_{0}}^{\text{opt}}(X)\right\}=-\left[2\cdot 1\left\{A=d_{\theta_{0}}^{\text{opt}}(X)\right\}-1\right]$, which implies that

$$\sqrt{n}E\left(X\left[1\left\{A={\hat{d}}_{{\hat{\theta }}_{n},n}(X)\right\}-1\left\{A=d_{\theta_{0}}^{\text{opt}}(X)\right\}\right]\right)\;=-\sqrt{n}E\left[X\left\{2P_{\beta_{0}}(X)-1\right\}1\left\{{\hat{d}}_{{\hat{\theta }}_{n},n}(X)\neq d_{\theta_{0}}^{\text{opt}}(X)\right\}\right]\;=-\sqrt{n}E\{X\{2P_{\beta_{0}}(X)-1\}(1\left[0\leq D_{\theta_{0}}(X)<-\left\{{\hat{D}}_{{\hat{\theta }}_{n},n}(X)-D_{\theta_{0}}(X)\right\}\right]\;=-\sqrt{n}E\{X\{2P_{\beta_{0}}(X)-1\}(1\left[0\leq D_{\theta_{0}}(X)<-\left\{{\hat{D}}_{{\hat{\theta }}_{n},n}(X)-D_{\theta_{0}}(X)\right\}\right]+1\left[-\left\{{\hat{D}}_{{\hat{\theta }}_{n},n}(X)-D_{\theta_{0}}(X)\right\}\leq D_{\theta_{0}}(X)<0\right])\}$$

$$=-\sqrt{n}E\left[X\left\{2P_{\beta_{0}}(X)-1\right\}\left(\int_{0}^{-\left({\hat{D}}_{{\hat{\theta }}_{n}}-D_{\theta_{0}}\right)}f_{0}(u)du+\int_{-\left({\hat{D}}_{{\hat{\theta }}_{n}}-D_{\theta_{0}}\right)}^{0}f_{0}(u)du\right)\|D_{\theta_{0}}(X)|\leq \epsilon_{n}\right]\times \left(1+o_{P}(1)\right)\;=-E\left[X\left\{2P_{\beta_{0}}(X)-1\right\}\cdot \left|\sqrt{n}\left\{{\hat{D}}_{{\hat{\theta }}_{n},n}(X)-D_{\theta_{0}}(X)\right\}\right||D_{\theta_{0}}(X)=0\right]f_{0}+o_{P}(1),$$

for some sequence *ϵ*_*n*_ converging down to zero slowly enough. The second to last equality holds by the fact that ${\left\|{\hat{D}}_{{\hat{\theta }}_{n},n}(x)-D_{\theta_{0}}(x)\right\|}_{X}=o_{P}(1)$ and Assumption 10 yields that the distribution of ***X*** converges to a random variable independent of $D_{\theta_{0}}(X)$ conditional on $\left|D_{\theta_{0}}(X)\right|\leq \epsilon_{n}$, as *n* goes to ∞.

Note that

$$\sqrt{n}\left\{{\hat{D}}_{{\hat{\theta }}_{n},n}(X)-D_{\theta_{0}}(X)\right\}=\sqrt{n}[\omega \left(X;{\hat{\theta }}_{n}\right)\left\{{\hat{R}}_{Y,n}(X)-R_{Y}(X)\right\}+\left\{1-\omega \left(X;{\hat{\theta }}_{n}\right)\right\}\left\{{\hat{R}}_{Z,n}(X)-R_{Z}(X)\right\}]+\sqrt{n}\left\{\omega \left(X;{\hat{\theta }}_{n}\right)-\omega \left(X;\theta_{0}\right)\right\}\left\{R_{Y}(X)-R_{Z}(X)\right\}\;=\omega \left(X;\theta_{0}\right)\phi_{Y}{\left(X\right)}^{⊤}Z_{Y,n}+\left\{1-\omega \left(X;\theta_{0}\right)\right\}\phi_{Z}{\left(X\right)}^{⊤}Z_{Z,n}+{\dot{\omega }}_{\theta_{0}}(X)\left\{R_{Y}(X)-R_{Z}(X)\right\}\sqrt{n}\left({\hat{\theta }}_{n}-\theta_{0}\right)\left\{1+o_{P}(1)\right\}\;=O_{P}\left(1+\sqrt{n}\|{\hat{\theta }}_{n}-\theta_{0}\|\right),$$

thus, $\left\|{\hat{u}}_{n}\left({\hat{\theta }}_{n}\right)\right\|=O_{P}\left(1+\sqrt{n}\left\|{\hat{\theta }}_{n}-\theta_{0}\right\|\right)$. Letting $v_{n}\left({\hat{\theta }}_{n},\beta_{*}\right)=n^{-1/2}{\hat{u}}_{n}{\left({\hat{\theta }}_{n}\right)}^{⊤}I_{n}^{-1}\left(\beta_{*}\right)\cdot {\hat{u}}_{n}\left({\hat{\theta }}_{n}\right)$,

$$0\leq n^{-1/2}\left\{{\hat{l}}_{n}\left({\hat{\theta }}_{n},{\hat{\beta }}_{n}\right)-{\hat{l}}_{n}\left(\theta_{0},\beta_{0}\right)\right\}\;=n^{-1/2}\sum_{i=1}^{n}X_{i}^{⊤}\beta_{0}\left[1\left\{A_{i}={\hat{d}}_{{\hat{\theta }}_{n},n}(X)\right\}-1\left\{A_{i}={\hat{d}}_{\theta_{0},n}(X)\right\}\right]+v_{n}\left({\hat{\theta }}_{n},\beta_{*}\right)/2\;=n^{1/2}E\left(X^{⊤}\beta_{0}\left[1\left\{A={\hat{d}}_{{\hat{\theta }}_{n},n}(X)\right\}-1\left\{A={\hat{d}}_{\theta_{0},n}(X)\right\}\right]\right)+o_{P}\left(1+\sqrt{n}\left\|{\hat{\theta }}_{n}-\theta_{0}\right\|\right)\;=n^{1/2}E\left(X^{⊤}\beta_{0}\left[1\left\{A={\hat{d}}_{{\hat{\theta }}_{n},n}(X)\right\}-1\left\{A=d_{\theta_{0}}^{\text{opt}}(X)\right\}\right]\right)-n^{1/2}E\left(X^{⊤}\beta_{0}\left[1\left\{A={\hat{d}}_{\theta_{0},n}(X)\right\}-1\left\{A=d_{\theta_{0}}^{\text{opt}}(X)\right\}\right]\right)+o_{P}\left(1+\sqrt{n}\left\|{\hat{\theta }}_{n}-\theta_{0}\right\|\right)\;=-E\left[X^{⊤}\beta_{0}\left\{2P_{\beta_{0}}(X)-1\right\}\cdot \left|\sqrt{n}\left\{{\hat{D}}_{{\hat{\theta }}_{n},n}(X)-D_{\theta_{0}}(X)\right\}\right||D_{\theta_{0}}(X)=0\right]f_{0}r_{n}+O_{P}(1)+o_{P}\left(1+\sqrt{n}\left\|{\hat{\theta }}_{n}-\theta_{0}\right\|\right)\;\leq -E\left[X^{⊤}\beta_{0}\left\{2P_{\beta_{0}}(X)-1\right\}\cdot \left|\left\{R_{Y}(X)-R_{Z}(X)\right\}{\dot{\omega }}_{\theta_{0}}{\left(X\right)}^{⊤}\sqrt{n}\left({\hat{\theta }}_{n}-\theta_{0}\right)\right||D_{\theta_{0}}(X)=0\right]f_{0}r_{n}+O_{P}(1)+o_{P}\left(1+\sqrt{n}\left\|{\hat{\theta }}_{n}-\theta_{0}\right\|\right)\;\leq -\delta_{1}^{2}\left(\frac{\exp\left(\delta_{1}\right)-1}{\exp\left(\delta_{1}\right)+1}\right)\underset{t\in S^{d}}{\inf}\left\{\mathrm{Pr}\left[\left|X^{⊤}\beta_{0}\right|\geq \delta_{1},\left|\left\{R_{Y}(X)-R_{Z}(X)\right\}{\dot{\omega }}_{\theta_{0}}{\left(X\right)}^{⊤}t\right|\geq \delta_{1}|D_{\theta_{0}}(X)=0\right]\right\}\times \sqrt{n}\left\|{\hat{\theta }}_{n}-\theta_{0}\right\|f_{0}r_{n}+O_{P}(1)+o_{P}\left(1+\sqrt{n}\left\|{\hat{\theta }}_{n}-\theta_{0}\right\|\right)\;\leq -\delta_{2}\delta_{1}^{2}\left(\frac{\exp\left(\delta_{1}\right)-1}{\exp\left(\delta_{1}\right)+1}\right)\sqrt{n}\left\|{\hat{\theta }}_{n}-\theta_{0}\right\|\left\{1+o_{P}(1)\right\}+O_{P}(1)+o_{P}\left(1+\sqrt{n}\left\|{\hat{\theta }}_{n}-\theta_{0}\right\|\right),$$

where *r*_*n*_ = 1 + *o*_*P*_ (1). This implies that $\sqrt{n}\left\|{\hat{\theta }}_{n}-\theta_{0}\right\|=O_{P}(1)$. From the above calculations, and the fact that the arg min or arg max of a function *t* ↦ *g*(*t*) does not change if we add a term that is invariant in *t*, we obtain that ${\hat{\theta }}_{n}=\text{arg }{\text{min}}_{t\in {\mathbb{R}}^{d}}M_{n}(t)$, where

$$M_{n}(t)=n^{-1/2}\sum_{i=1}^{n}X_{i}^{⊤}\beta_{0}\left[1\left\{A_{i}={\hat{d}}_{t,n}(X)\right\}-1\left\{A_{i}=d_{\theta_{0}}^{\text{opt}}(X)\right\}\right]+v_{n}\left(t,\beta_{*}\right)/2.$$

Let $M(u)=\beta_{0}^{⊤}k_{0}\left(Z_{Y},Z_{Z},u\right)$ and $U=\text{arg }{\text{min}}_{u\in {\mathbb{R}}^{d}}M(u)$. We will show that $M_{n}(\theta_{0}+u/\sqrt{n})⇝M(u)$ in *ℓ*^∞^(*K*) for any compact subset *K* of ${\mathbb{R}}^{d}$. Then, it will follow from the argmax Theorem (See chapter 14 of Kosorok, 2008) that ${\tilde{U}}_{n}⇝U$, where ${\tilde{U}}_{n}=\text{arg }{\text{min}}_{u\in {\mathbb{R}}^{d}}M_{n}\left(\theta_{0}+u/\sqrt{n}\right)$. Let $h_{n}(u)=\theta_{0}+u/\sqrt{n}$. Similar arguments along with Assumptions 11 and 10, yield that, for any compact $K\subset {\mathbb{R}}^{d}$,

$$\underset{u\in K}{\text{arg min}}M_{n}\left\{h_{n}(u)\right\}\;=\underset{u\in K}{\text{arg min}}n^{1/2}E\left(X^{⊤}\beta_{0}\left[1\left\{A={\hat{d}}_{h_{n}(u),n}(X)\right\}-1\left\{A=d_{\theta_{0}}^{\text{opt}}(X)\right\}\right]\right)+o_{P}(1)\;=\underset{u\in K}{\text{arg min}}n^{1/2}E\{X^{⊤}\beta_{0}\{2P_{\beta_{0}}(X)-1\}(1[-\left\{{\hat{D}}_{h_{n}(u),n}(X)-D_{\theta_{0}}(X)\right\}\leq D_{\theta_{0}}(X)<0]+1\left[0\leq D_{\theta_{0}}(X)<-\left\{{\hat{D}}_{h_{n}(u),n}(X)-D_{\theta_{0}}(X)\right\}\right])\}+o_{P}(1)\;=\underset{u\in K}{\text{arg min}}E[X^{⊤}\beta_{0}\left\{2P_{\beta_{0}}(X)-1\right\}\left|\sqrt{n}\left\{{\hat{D}}_{h_{n}(u),n}(X)-D_{\theta_{0}}(X)\right\}\right||D_{\theta_{0}}(X)=0]f_{0}+o_{P}(1),$$

However,

$$\sqrt{n}\left\{{\hat{D}}_{h_{n}(u),n}(X)-D_{\theta_{0}}(X)\right\}⇝\omega \left(X;\theta_{0}\right)\phi_{Y}{\left(X\right)}^{⊤}Z_{Y}+\left\{1-\omega \left(X;\theta_{0}\right)\right\}\phi_{Z}{\left(X\right)}^{⊤}Z_{Z}+R_{0}(X){\dot{\omega }}_{\theta_{0}}{\left(X\right)}^{⊤}u$$

uniformly over X when ***X*** has its conditional distribution given $D_{\theta_{0}}(X)=0$. By applying continuous mapping theorem, this implies that $M_{n}(\theta +u/\sqrt{n})⇝M(u)$ in *ℓ*^∞^(*K*) as desired and thus ${\tilde{U}}_{n}⇝U$. It is straightforward to verify the remaining conclusions of the theorem using previous arguments.■

**Proof** [Proof of Theorem 14] Using the assumptions, the fact that both $\sqrt{n}\left({\hat{\theta }}_{n}-\theta_{0}\right)=O_{P}(1)$ and $\sqrt{n}\left({\hat{\beta }}_{n}-\beta_{0}\right)=O_{P}(1)$, and standard arguments, we obtain that, for any compact $K_{1}\subset {\mathbb{R}}^{q}$, ${\text{sup}}_{{\left(Z_{Y}^{⊤},Z_{Z}^{⊤}\right)}^{⊤}\in K_{1}}{\left\|{\tilde{T}}_{n}\left\{x,{\tilde{Z}}_{Y}\left(Z_{Y},Z_{Z}\right),{\tilde{Z}}_{Z}\left(Z_{Y},Z_{Z}\right)\right\}-T_{0}\left(x,Z_{Y},Z_{Z}\right)\right\|}_{X}=O_{P}(1)$, where

$$\left(\begin{array}{c} {\tilde{Z}}_{Y}\left(Z_{Y},Z_{Z}\right)\\ {\tilde{Z}}_{Z}\left(Z_{Y},Z_{Z}\right) \end{array}\right)={\hat{\Sigma }}_{1}^{1/2}\Sigma_{1}^{-1/2}\left(\begin{array}{c} Z_{Y}\\ Z_{Z} \end{array}\right),$$

Σ_1_ is the upper left *q* × *q* block of Σ_0_, and also *T*_0_(***x***, *Z*_*Y*_, *Z*_*Z*_) = *ω*(***x***; *θ*_0_)*ϕ*_*Y*_ (***x***)^⊤^*Z*_*Y*_ + {1 − *ω*(***x***; *θ*_0_)}*ϕ*_*Z*_(***x***)^⊤^*Z*_*Z*_. Furthermore,

$${\left\|\left\{{\hat{R}}_{Y,n}(x)-{\hat{R}}_{Z,n}(x)\right\}{\dot{\omega }}_{{\hat{\theta }}_{n}}{\left(x\right)}^{⊤}u-\left\{R_{Y}(x)-R_{Z}(x)\right\}{\dot{\omega }}_{\theta_{0}}{\left(x\right)}^{⊤}u\right\|}_{X}\;\leq {\left\|\left\|\left\{{\hat{R}}_{Y,n}(x)-{\hat{R}}_{Z,n}(x)\right\}{\dot{\omega }}_{{\hat{\theta }}_{n}}(x)-\left\{R_{Y}(x)-R_{Z}(x)\right\}{\dot{\omega }}_{\theta_{0}}(x)\right\|\right\|}_{X}\cdot \|u\|\;=O_{P}\left(n^{-1/2}\right)\|u\|$$

${\left\|{\hat{D}}_{{\hat{\theta }}_{n},n}(x)-D_{\theta_{0}}(x)\right\|}_{X}=O_{P}\left(n^{-1/2}\right)$, and ${\left\|P_{{\hat{\beta }}_{n}}(x)-P_{\beta_{0}}(x)\right\|}_{X}=O_{P}\left(n^{-1/2}\right)$. Thus,

(9)
$$\underset{{\left(Z_{Y}^{⊤},Z_{Z}^{⊤}\right)}^{⊤}\in K_{1}}{\text{sup}}E_{n}\left[\|X\|\cdot \left|\left\{2P_{{\hat{\beta }}_{n}}(X)-1\right\}{\tilde{T}}_{n}\left\{X,{\tilde{Z}}_{Y}\left(Z_{Y},Z_{Z}\right),{\tilde{Z}}_{Z}\left(Z_{Y},Z_{Z}\right)\right\}\right|\frac{1}{h_{n}}\phi_{0}\left\{\frac{{\hat{D}}_{{\hat{\theta }}_{n},n}(X)}{h_{n}}\right\}\right]\leq O_{P}(1)E_{n}\left[\frac{1}{h_{n}}\phi_{0}\left\{\frac{{\hat{D}}_{{\hat{\theta }}_{n},n}(X)}{h_{n}}\right\}\right].$$

However,

$$E_{n}\left(\frac{1}{h_{n}}\left[\phi_{0}\left\{\frac{{\hat{D}}_{{\hat{\theta }}_{n},n}(X)}{h_{n}}\right\}-\phi_{0}\left\{\frac{D_{\theta_{0}}(X)}{h_{n}}\right\}\right]\right)\;=E_{n}\left[\frac{1}{h_{n}^{3}}\int_{0}^{1}\left\{\left(1-s)D_{\theta_{0}}(X)+s{\hat{D}}_{{\hat{\theta }}_{n},n}(X\right)\right\}\phi_{0}\left\{\frac{\left(1-s)D_{\theta_{0}}(X)+s{\hat{D}}_{{\hat{\theta }}_{n},n}(X\right)}{h_{n}}\right\}\cdot \text{d}s\left\{{\hat{D}}_{{\hat{\theta }}_{n},n}(X)-D_{\theta_{0}}(X)\right\}\right]\;=O_{P}\left(\frac{1}{h_{n}^{3}n^{1/2}}\right)\cdot E_{n}\left[\int_{0}^{1}\left\{\left(1-s)D_{\theta_{0}}(X)+s{\hat{D}}_{{\hat{\theta }}_{n},n}(X\right)\right\}\phi_{0}\left\{\frac{\left(1-s)D_{\theta_{0}}(X)+s{\hat{D}}_{{\hat{\theta }}_{n},n}(X\right)}{h_{n}}\right\}\text{d}s\right]\;=O_{P}\left(\frac{1}{h_{n}^{3}n^{1/2}}\right)O_{P}\left(h_{n}\right)=O_{P}\left(\frac{1}{h_{n}^{2}n^{1/2}}\right)=o_{P}(1),$$

since $\left|u\phi_{0}(u)\right|\leq {\left(2\pi \right)}^{-1/2}e^{-1}<\infty$. Now, since $\;E\left[h_{n}^{-1}\phi_{0}\left\{D_{\theta_{0}}(X)/h_{n}\right\}\right]\overset{P}{\to }f_{0}$, we have that (9) is equal to *O*_*P*_ (1). Thus, if $\left\|u_{n}\right\|\to \infty$,

(10)
$${\hat{\beta }}_{n}^{⊤}{\tilde{k}}_{n}\left({\tilde{Z}}_{Y},{\tilde{Z}}_{Z},u_{n}\right)\geq E_{n}\left[{\hat{\beta }}_{n}^{⊤}X\left\{2P_{{\hat{\beta }}_{n}}(X)-1\right\}\cdot \left|\left\{{\hat{R}}_{Y,n}(X)-{\hat{R}}_{Z,n}(X)\right\}{\dot{\omega }}_{{\hat{\theta }}_{n}}{\left(X\right)}^{⊤}u_{n}\right|\cdot \frac{1}{h_{n}}\phi_{0}\left\{\frac{{\hat{D}}_{{\hat{\theta }}_{n},n}(X)}{h_{n}}\right\}\right]-O_{P}(1),$$

where the *O*_*P*_ (1) is uniform over *K*_1_. Thus, up to the *O*_*P*_ (1) added on the right-hand side,

$$(10)\geq \left\|u_{n}\right\|\cdot \underset{t\in S^{d}}{\text{inf}}E_{n}\left[{\hat{\beta }}_{n}^{⊤}X\left\{2P_{{\hat{\beta }}_{n}}(X)-1\right\}\left|\left\{{\hat{R}}_{Y,n}(X)-{\hat{R}}_{Z,n}(X)\right\}{\dot{\omega }}_{{\hat{\theta }}_{n}}{\left(X\right)}^{⊤}t\right|\frac{1}{h_{n}}\phi_{0}\left\{\frac{{\hat{D}}_{{\hat{\theta }}_{n},n}(X)}{h_{n}}\right\}\right]\;\geq u_{n}\left(o_{P}(1)+\underset{t\in S^{d}}{\text{inf}}E\left[\beta_{0}^{⊤}X\left\{2P_{\beta_{0}}(X)-1\right\}\left|\left\{R_{Y}(X)-R_{Z}(X)\right\}{\dot{\omega }}_{\theta_{0}}{\left(X\right)}^{⊤}t\right|\frac{1}{h_{n}}\phi_{0}\left\{\frac{D_{\theta_{0}}(X)}{h_{n}}\right\}\right]\right)\;=\left\|u_{n}\right\|\cdot \left(o_{P}(1)+\underset{t\in S^{d}}{\text{inf}}E\left[\beta_{0}^{⊤}X\left\{2P_{\beta_{0}}(X)-1\right\}\left|\left\{R_{Y}(X)-R_{Z}(X)\right\}{\dot{\omega }}_{\theta_{0}}{\left(X\right)}^{⊤}t\right||D_{\theta_{0}}(X)=0\right]f_{0}\right)\;\geq \left\|u_{n}\right\|\left[o_{P}(1)+\delta_{2}\delta_{1}^{2}\left\{\frac{\text{exp}\left(\delta_{1}\right)-1}{\text{exp}\left(\delta_{1}\right)+1}\right\}\right],$$

with the expectation over ***X***. Let ${\hat{U}}_{n}\left(Z_{Y},Z_{Z}\right)=\text{arg }{\text{min}}_{u\in {\mathbb{R}}^{d}}{\hat{\beta }}_{n}^{⊤}{\tilde{k}}_{n}\left\{{\tilde{Z}}_{Y}\left(Z_{Y},Z_{Z}\right),{\tilde{Z}}_{Z}\left(Z_{Y},Z_{Z}\right),u\right\}$, where, if the arg min set has more than one element, one can be chosen randomly or algorithmically. Since the *O*_*P*_ (1) above is uniform over *K*_1_, we conclude that

(11)
$$\underset{{\left(Z_{Y}^{⊤},Z_{Z}^{⊤}\right)}^{⊤}\in K_{1}}{\text{sup}}\left\|{\hat{U}}_{n}\left(Z_{Y},Z_{Z}\right)\right\|=O_{P}(1).$$

Now, let *K*_2_ be any compact subset of ${\mathbb{R}}^{d}$. Previous and standard arguments give us that ${\text{sup}}_{{\left(Z_{Y}^{⊤},Z_{Z}^{⊤}\right)}^{⊤}\in K_{1}}{\text{sup}}_{u\in K_{2}}\left\|{\tilde{k}}_{n}\left\{{\tilde{Z}}_{Y}\left(Z_{Y},Z_{Z}\right),{\tilde{Z}}_{Z}\left(Z_{Y},Z_{Z}\right),u\right\}-k_{0}\left(Z_{Y},Z_{Z},u\right)\right\|=o_{P}(1)$. Thus, we also have that

(12)
$$\underset{{\left(Z_{Y}^{⊤},Z_{Z}^{⊤}\right)}^{⊤}\in K_{1}}{\text{sup}}\underset{u\in K_{2}}{\text{sup}}\left\|{\hat{\beta }}_{n}^{⊤}{\tilde{k}}_{n}\left\{{\tilde{Z}}_{Y}\left(Z_{Y},Z_{Z}\right),{\tilde{Z}}_{Z}\left(Z_{Y},Z_{Z}\right),u\right\}-\beta_{0}^{⊤}k_{0}\left(Z_{Y},Z_{Z},u\right)\right\|=o_{P}(1).$$

Define $U_{0}\left(Z_{Y},Z_{Z}\right)=\text{arg }{\text{max}}_{u\in {\mathbb{R}}^{d}}\beta_{0}^{⊤}k_{0}\left(Z_{Y},Z_{Z},u\right)$. Previous arguments yield that

(13)
$$\underset{{\left(Z_{Y}^{⊤},Z_{Z}^{⊤}\right)}^{⊤}\in K_{1}}{\text{sup}}\left\|U_{0}\left(Z_{Y},Z_{Z}\right)\right\|=O(1).$$

By Assumption 11, the arg min for each ${\left(Z_{Y}^{⊤},Z_{Z}^{⊤}\right)}^{⊤}\in K_{1}$ is unique. Fix *ϵ >* 0. By (11), there exists an *m*_2_ *<* ∞ such that $\text{Pr}\left({\text{sup}}_{{\left(Z_{Y}^{⊤},Z_{Z}^{⊤}\right)}^{⊤}\in K_{1}}\left\|{\hat{U}}_{n}\left(Z_{Y},Z_{Z}\right)\right\|<m_{2}\right)\geq 1-\epsilon$ for all n large enough. By (13), we can enlarge *m*_2_ such that

$${\text{sup}}_{{\left(Z_{Y}^{⊤},Z_{Z}^{⊤}\right)}^{⊤}\in K_{1}}\left\|U_{0}\left(Z_{Y},Z_{Z}\right)\right\|<m_{2}<\infty .$$

We can also find an *m*_1_ *<* ∞ such that $K_{1}\subset K_{m_{1}}^{q}$ as defined in Corollary 17. It is straightforward to show that (1) and (3) in Corollary 17 are satisfied by $f(Z,u)=\beta_{0}^{⊤}k_{0}\left(Z_{Y},Z_{Z},u\right)$, where $Z={\left(Z_{Y}^{⊤},Z_{Z}^{⊤}\right)}^{⊤}$. Let $f_{n}(Z,u)={\hat{\beta }}_{n}^{⊤}{\tilde{k}}_{n}\left(Z_{Y},Z_{Z},u\right)$. Standard arguments and the given assumptions yield that there exists a *w*_1_ *<* ∞ such that ${\text{sup}}_{Z\in K_{m_{1}}^{q}}{\text{sup}}_{u\in K_{m_{2}}^{d}}\left|f_{n}(Z,u)\right|<w_{1}$ almost surely and

$$\underset{Z_{1},Z_{2}\in K_{m1}^{q}:\left\|Z_{1}-Z_{2}\right\|<\delta }{\text{sup}}{\left\|f_{n}\left(Z_{1},u\right)-f_{n}\left(Z_{2},u\right)\right\|}_{K_{m_{2}}^{d}}<w_{1}\delta$$

for all *δ >* 0 and all *n* ≥ 1 almost surely. Every subsequence in (12) has a further subsequence *n*″ on which the convergence in probability to zero can be replaced with almost sure convergence. Thus, (2) and (4) of Corollary 17 apply, using the fact that minimizing is equivalent to maximizing after a change in sign. Setting ${\hat{U}}_{n}^{*}\left(Z_{Y},Z_{Z}\right)=\text{arg }{\text{min}}_{u\in K_{m_{2}}^{d}}{\hat{\beta }}_{n}^{⊤}\;{\tilde{k}}_{n}\left\{{\tilde{Z}}_{Y}\left(Z_{Y},Z_{Z}\right),{\tilde{Z}}_{Z}\left(Z_{Y},Z_{Z}\right),u\right\}$, Corollary 17 now yields that

$$\underset{{\left(Z_{Y}^{⊤},Z_{Z}^{⊤}\right)}^{⊤}\in K_{m_{1}}^{q}}{\text{sup}}\left\|{\hat{U}}_{n^{\prime \prime }}^{*}\left(Z_{Y},Z_{Z}\right)-U_{0}\left(Z_{Y},Z_{Z}\right)\right\|\to 0$$

almost surely. Since this is true for every subsequence, we have that

$$\underset{{\left(Z_{Y}^{⊤},Z_{Z}^{⊤}\right)}^{⊤}\in K_{m_{1}}^{q}}{\text{sup}}\left\|{\hat{U}}_{n}^{*}\left(Z_{Y},Z_{Z}\right)-U_{0}\left(Z_{Y},Z_{Z}\right)\right\|\overset{P}{\to }0$$

as *n* → ∞. Note that, on *K*_2_, ${\hat{U}}_{n}^{*}\left(Z_{Y},Z_{Z}\right)={\hat{U}}_{n}\left(Z_{Y},Z_{Z}\right)$ for all ${\left(Z_{Y}^{⊤},Z_{Z}^{⊤}\right)}^{⊤}\in K_{m_{1}}^{q}$. Hence,

$$\underset{n\to \infty }{\text{lim sup}}\text{Pr }\left\{\underset{{\left(Z_{Y}^{⊤},Z_{Z}^{⊤}\right)}^{⊤}\in K_{m_{1}}^{q}}{\text{sup}}\left\|{\hat{U}}_{n}\left(Z_{Y},Z_{Z}\right)-U_{0}\left(Z_{Y},Z_{Z}\right)\right\|>\epsilon \right\}\;\leq \underset{n\to \infty }{\text{lim sup }}\left[\text{Pr }\left\{{\hat{U}}_{n}\left(Z_{Y},Z_{Z}\right)\in K_{2},\underset{{\left(Z_{Y}^{⊤},Z_{Z}^{⊤}\right)}^{⊤}\in K_{m_{1}}^{q}}{\text{sup}}\left\|{\hat{U}}_{n}^{*}\left(Z_{Y},Z_{Z}\right)-U_{0}\left(Z_{Y},Z_{Z}\right)\right\|\geq \epsilon \right\}+\text{Pr}\left\{{\hat{U}}_{n}\left(Z_{Y},Z_{Z}\right)\in K_{2}^{c}\right\}\right]\;\leq \epsilon .$$

Since *ϵ* was arbitrary, we obtain that

$$\underset{{\left(Z_{Y}^{⊤},Z_{Z}^{⊤}\right)}^{⊤}\in K_{m_{1}}^{q}}{\text{sup}}\left\|{\hat{U}}_{n}\left(Z_{Y},Z_{Z}\right)-U_{0}\left(Z_{Y},Z_{Z}\right)\right\|=o_{P}(1).$$

Let $B_{L}(B)$ be the space of all Lipschitz continuous functions mapping B→ℝ which are bounded by 1 and which have Lipschitz constant 1. Let $E_{Z}$ be expectation with respect to $Z_{0}^{*}={\left(Z_{Y}^{*},Z_{Z}^{*},Z_{A}^{*⊤}\right)}^{⊤}\sim N\left(0,\Sigma_{0}\right)$. Let $B_{0}\left(Z_{0}^{*}\right)=I_{0}^{-1}\left[Z_{A}^{*}-k_{0}\left\{Z_{Y}^{*},Z_{Z}^{*},\;{\tilde{U}}_{n}\left(Z_{0}^{*}\right)\right\}\right]$ and let $f\in B_{L}\left({\mathbb{R}}^{d+p}\right)$. Then, using ${\tilde{U}}_{n}$ and ${\tilde{B}}_{n}$ as defined in the statement of the theorem,

$$\left|E_{Z}\left[f\left\{{\tilde{U}}_{n}\left(Z_{0}^{*}\right),{\tilde{B}}_{n}\left(Z_{0}^{*}\right)\right\}-f\left\{U_{0}\left(Z_{0}^{*}\right),B_{0}\left(Z_{0}^{*}\right)\right\}\right]\right|\;\leq \left|E_{Z}\left[f\left\{{\tilde{U}}_{n}\left(Z_{0}^{*}\right),{\tilde{B}}_{n}\left(Z_{0}^{*}\right)\right\}-f\left\{U_{0}\left(Z_{0}^{*}\right),{\tilde{B}}_{n}\left(Z_{0}^{*}\right)\right\}\right]\right|+\left|E_{Z}\left[f\left\{U_{0}\left(Z_{0}^{*}\right),{\tilde{B}}_{n}\left(Z_{0}^{*}\right)\right\}-f\left\{U_{0}\left(Z_{0}^{*}\right),B_{0}\left(Z_{0}^{*}\right)\right\}\right]\right|\;=\left|E_{Z}\left[f_{1}\left\{{\tilde{U}}_{n}\left(Z_{0}^{*}\right)\right\}-f_{1}\left\{U_{0}\left(Z_{0}^{*}\right)\right\}\right]\right|+\left|E_{Z}\left[f_{2}\left\{{\tilde{B}}_{n}\left(Z_{0}^{*}\right)\right\}-f_{2}\left\{B_{0}\left(Z_{0}^{*}\right)\right\}\right]\right|$$

for some other $f_{1}\in B_{L}\left({\mathbb{R}}^{d}\right)$ and $f_{2}\in B_{L}\left({\mathbb{R}}^{p}\right)$. Hence,

$$\underset{f\in B_{L}\left({\mathbb{R}}^{d+p}\right)}{\text{sup}}\left|E_{Z}f\left\{{\tilde{U}}_{n}\left(Z_{0}^{*}\right),{\tilde{B}}_{n}\left(Z_{0}^{*}\right)\right\}-E_{Z}f\left\{U_{0}\left(Z_{0}^{*}\right),B_{0}\left(Z_{0}^{*}\right)\right\}\right|\;\leq \underset{f\in B_{L}\left({\mathbb{R}}^{d}\right)}{\text{sup}}\left|E_{Z}f\left\{{\tilde{U}}_{n}\left(Z_{0}^{*}\right)\right\}-E_{Z}f\left\{U_{0}\left(Z_{0}^{*}\right)\right\}\right|+\underset{f\in B_{L}\left({\mathbb{R}}^{p}\right)}{\text{sup}}\left|E_{Z}f\left\{{\tilde{B}}_{n}\left(Z_{0}^{*}\right)\right\}-E_{Z}f\left\{B_{0}\left(Z_{0}^{*}\right)\right\}\right|\;=A_{n}+B_{n},$$

where we define both $A_{n}={\text{sup}}_{f\in B_{L}\left({\mathbb{R}}^{d}\right)}\left|E_{Z}f\left\{{\tilde{U}}_{n}\left(Z_{0}^{*}\right)\right\}-E_{Z}f\left\{U_{0}\left(Z_{0}^{*}\right)\right\}\right|$ and

$$B_{n}={\text{sup}}_{f\in B_{L}\left({\mathbb{R}}^{p}\right)}\left|E_{Z}f\left\{{\tilde{B}}_{n}\left(Z_{0}^{*}\right)\right\}-E_{Z}f\left\{B_{0}\left(Z_{0}^{*}\right)\right\}\right|.$$

Fix some compact $K_{1}\subset {\mathbb{R}}^{q}$ such that $\text{Pr}\left\{{\left(Z_{Y}^{*⊤},Z_{Z}^{*}\right)}^{⊤}\in K_{1}\right\}\geq 1-\epsilon$. Then,

$$\underset{f\in B_{L}\left({\mathbb{R}}^{d}\right)}{\text{sup}}\left|E_{Z}\left[f\left\{{\tilde{U}}_{n}\left(Z_{0}^{*}\right)\right\}-E_{Z}f\left\{U_{0}\left(Z_{0}^{*}\right)\right\}\right]\right|\leq \underset{f\in B_{L}\left({\mathbb{R}}^{d}\right)}{\text{sup}}\left|E_{Z}1\left(Z_{0}^{*}\in K_{1}\right)f\left\{{\tilde{U}}_{n}\left(Z_{0}^{*}\right)\right\}-E_{Z}1\left(Z_{0}^{*}\in K_{1}\right)f\left\{U_{0}\left(Z_{0}^{*}\right)\right\}\right|+2E_{Z}1\left(Z_{0}^{*}\in K_{1}\right)\;=o_{P}(1)+2\epsilon ,$$

which implies that *A*_*n*_ = *o*_*P*_ (1) since was arbitrary. For $K_{2}\subset {\mathbb{R}}^{q+p}$ such that $\text{Pr}\left(Z_{0}^{*}\in K_{2}\right)\geq 1-\epsilon$, previous arguments yield that

$${\text{sup}}_{Z_{0}^{*}\in K_{2}}\left\|{\tilde{B}}_{n}\left(Z_{0}^{*}\right)-B_{0}\left(Z_{0}^{*}\right)\right\|=o_{P}(1).$$

As before, we can argue that *B*_*n*_ = *o_P_* (1) + 2*ϵ*, which implies that *B*_*n*_ = *o*_*P*_ (1) since *ϵ* was arbitrary. The result follows.■

**Theorem 16** *Let H be a compact set in a metric space with metric d and let F be a compact subset of C*[*H*] *with respect to the uniform norm*, ∥ · ∥_*H*_. *For each f∈F, let $u(f)=\text{arg }{\text{max}}_{h\in H}f(h)$, where, when the arg max is not unique, we select one element of the arg max set either randomly or algorithmically. Suppose also that there exists a closed $F_{1}\subset F$ for which each $f\in F_{1}$ has a unique maximum. Then*,

$$\underset{\delta ↓0}{\text{lim}}\underset{f\in F_{1}}{\text{sup}}\underset{g\in F:\|f-g{\|}_{H}<\delta }{\text{sup}}d\{u(f),u(g)\}=0.$$

**Proof** [Proof of Theorem 16] Fix *ϵ >* 0. For each $f\in F_{1}$, there exists *δ*_*f*_ *>* 0 such that

$$\underset{h\in H\cap B_{\epsilon }{\left\{u(f)\right\}}^{c}}{\text{sup}}f(h)<f\{u(f)\}-2\delta_{f},$$

where *B*_*ϵ*_(*u*) is the open *d*-ball of radius *ϵ* around *u*. This follows since the compactness of F ensures that all f∈F are continuous. Let g∈F be such that ∥*f* – *g*∥_*H*_ *< δ*_*f*_. Then, *f* {*u*(*g*)} *> g* {*u*(*g*)} − *δ*_*f*_ ≥ *g* {*u*(*f*)} − *δ*_*f*_ *> f* {*u*(*f*)} − 2*δ*_*f*_, which implies that *d*{*u*(*g*), *u*(*f*)} < *ϵ*. We have that $\cup_{f\in F_{1}}\left\{g\in F:\|g-f{\|}_{H}<\delta_{f}\right\}$ is an open cover of $F_{1}$. Since $F_{1}$ is compact, there exists a set $F_{1}^{\epsilon }$ such that $F_{1}^{\epsilon }$ is finite and

$$\cup_{f\in F_{1}^{\epsilon }}\left\{g\in F:\|g-f{\|}_{H}<\delta_{f}\right\}$$

still covers $F_{1}$. Let $\left\{f_{n}\right\}\in F$ and $\left\{g_{n}\right\}\in F$ be sequences such that ∥*f*_*n*_ − *g*_*n*_∥ → 0. By compactness, every subsequence has a further subsequence *n*″ such that $f_{n^{\prime \prime }}\to f_{0}\in F_{1}$ and $g_{n^{\prime \prime }}\to g_{0}\in F$ so that both *f*_0_ and *g*_0_ are in a set of the form $\left\{g\in F:\|g-f{\|}_{H}<\delta_{f}\right\}$ for some $f\in F_{1}^{\epsilon }$. This implies that *d*{*u*(*g*_0_), *u*(*f*_0_)} < *ϵ*. Since the subsequence was arbitrary, we have that lim sup_*n*→∞_*d*{*u*(*g*_*n*_), *u*(*f*_*n*_)} ≤ *ϵ*. Since *ϵ* was arbitrary, we now have that lim sup_*n*→∞_*d*{*u*(*g*_*n*_), *u*(*f*_*n*_)} = 0 for any sequences $\left\{f_{n}\right\}\in F_{1}$ and $\left\{g_{n}\right\}\in F$ such that ∥*f*_*n*_ − *g*_*n*_∥ → 0. This proves the result.■

**Corollary 17** *For m*_1_ *<* ∞, *let $K_{m_{1}}^{q}=\left\{z\in {\mathbb{R}}^{q}:\|z\|\leq m_{1}\right\}$. Let* (*z*, *u*) ↦ *f*(*z*, *u*) *and* (*z*, *u*) ↦ *f*_*n*_(*z*, *u*) *be a fixed function and a sequence of functions, respectively, from* $K_{m_{1}}^{q}\times {\mathbb{R}}^{d}$ *to* ℝ. *Suppose there exists m*_2_ *<* ∞ *such that for each $z\in K_{m_{1}}^{q}$, $u(z)=\text{arg }{\text{max}}_{u\in {\mathbb{R}}^{d}}f(z,u)<m_{2}$ and is uniquely defined. Suppose also that $u_{n}(z)=\text{arg }{\text{max}}_{u\in {\mathbb{R}}^{d}}f_{n}(z,u)<m_{2}$ for all n large enough, where we allow the arg max to be non-unique, but we randomly or algorithmically select one element from the arg max set. Define* $K_{m_{2}}^{d}$ *similarly to $K_{m_{1}}^{q}$ and assume that*

1. ${\text{sup}}_{z\in K_{m_{1}}^{q}}{\text{sup}}_{u\in K_{m_{2}}^{d}}|f(z,u)|<\infty$
2. ${\text{lim sup}}_{n\to \infty }{\text{sup}}_{z\in K_{m_{1}}^{q}}{\text{sup}}_{u\in K_{m_{2}}^{d}}\left|f_{n}(z,u)\right|<\infty$
3. ${\text{lim}}_{\delta ↓0}{\text{sup}}_{z_{1},z_{2}\in K_{m_{1}}^{q}:\left\|z_{1}-z_{2}\right\|<\delta }{\left\|f\left(z_{1},\cdot \right)-f\left(z_{2},\cdot \right)\right\|}_{K_{m_{2}}^{d}}=0$
4. ${\text{lim}}_{\delta ↓0}{\text{sup}}_{z_{1},z_{2}\in K_{m_{1}}^{q}:\left\|z_{1}-z_{2}\right\|<\delta }{\left\|f_{n}\left(z_{1},\cdot \right)-f_{n}\left(z_{2},\cdot \right)\right\|}_{K_{m_{2}}^{d}}=0$

*for all n large enough. Then, provided* ${\text{sup}}_{z\in K_{m_{1}}^{q}}{\left\|f_{n}(z,\cdot )-f(z,\cdot )\right\|}_{K_{m_{2}}^{d}}\to 0$,

$$\underset{z\in K_{m_{1}}^{q}}{\text{sup}}\left\|u_{n}(z)-u(z)\right\|\to 0,$$

*as n* → ∞.

**Proof** [Corollary 17] By the Arzelà–Ascoli Theorem, there exists a compact *K* ⊂ *C*[*H*] for $H=K_{m_{2}}^{d}$, such that both *f*(*z*,·) ∈ *K* and *f*_*n*_(*z*,·) ∈ *K*, for all $z\in K_{m_{1}}^{q}$ and all *n* large enough. If we let $F_{1}=\left\{f(z,\cdot ):z\in K_{m_{1}}^{q}\right\}$, we can directly apply Theorem 16 to obtain that

$$\underset{\delta ↓0}{\text{lim}}\underset{z\in K_{m_{1}}^{q}}{\text{sup}}\underset{g\in K:\|g-f(z,\cdot ){\|}_{H}<\delta }{\text{sup}}\|u(g)-u\{f(z,\cdot )\}{\|}_{H}=0.$$

Because ${\text{sup}}_{z\in K_{m_{1}}^{q}}{\left\|f_{n}(z,\cdot )-f(z,\cdot )\right\|}_{K_{m_{2}}^{d}}<\delta$ for all *n* large enough, the result follows.■

**Proof** [Remark 6.] We require estimation be restricted to parameters (*β*, *θ*) which satisfy *P*_*β*_ {*A* = *d*_*θ*_(***X***)|***X***} *>* 1/2 with probability one. Suppose towards a contradiction that such a set of parameters also satisfies

$$P_{\beta }{\left(X\right)}^{1}\left\{A=d_{\theta }(X)\right\}{\left\{1-P_{\beta }(X)\right\}}^{1-1}\left\{A=d_{\theta }(X)\right\}=P_{\beta_{0}}{\left(X\right)}^{1\left\{A=d_{\theta_{0}}(X)\right\}}{\left\{1-P_{\beta_{0}}(X)\right\}}^{1-1\left\{A=d_{\theta_{0}}(X)\right\}}\text{ a}.\text{s}.,$$

where *P*_*β*_(***x***) = expit(***x***^**⊤**^*β*). For any (***x***, *a*) such that $a=d_{\theta }(x)\neq d_{\theta_{0}}(x)$ and it follows that $P_{\beta }(x)=P_{-\beta_{0}}(x)<P_{\beta_{0}}(x)=P_{-\beta }(x)$ which contradicts the restriction that the probability of recommending an optimal treatment exceeds 1/2. Thus, it follows that $d_{\theta }(x)=d_{\theta_{0}}(x)$ for almost all ***x***. This in turn implies that $P\beta (x)=P_{\beta_{0}}(x)$ for almost all ***x*** from which it follows that *β* = *β*_0_ provided $EXX^{⊤}$ is full rank.■

## Appendix B: Three or More Outcomes

Assume the available data consist of (***X***_*i*_, *A*_*i*_, *Y*_1,*i*_, …, *Y*_*K,i*_), *i* = 1, *…*, *n*, which comprise *n* independent and identically distributed copies of (***X***, *A*, *Y*_1_, …, *Y*_*K*_), where ***X*** and *A* are as defined previously, and *Y*_1_, …, *Y*_*K*_ are outcomes, with each outcome coded so that higher values are better. Assume there exists an unknown utility function *U* = *u*(*Y*_1_, …, *Y*_*K*_), where $u:{\mathbb{R}}^{K}\to \mathbb{R}$, such that *u*(*y*_1_, …, *y*_*K*_) quantifies the “goodness” of the outcome vector (*y*_1_, …, *y*_*K*_). As before, let *U**(*d*) be the potential utility under a treatment regime *d*. Let $d_{U}^{\text{opt}}$ be the optimal regime for the utility defined by *u*, i.e., $EU^{*}\left(d_{U}^{\text{opt}}\right)\geq EU^{*}(d)$ for any regime *d*. The goal is to estimate the utility function and the associated optimal regime in the presence of more than two outcomes.

To begin, we assume that the utility function is constant across patients and takes the form $u\left(y_{1},\ldots ,y_{K};\omega \right)=\sum_{k=1}^{K-1}\omega_{k}y_{k}+\left(1-\sum_{k=1}^{K-1}\omega_{k}\right)y_{K}$, where *ω* = (*ω*_1_, …, *ω*_*K*−1_) is a vector of parameters with $\sum_{k=1}^{K-1}\omega_{k}\leq 1$ and *ω*_*k*_ ≥ 0 for *k* = 1, …, *K* − 1. Thus, we assume that the utility function is a convex combination of the set of outcomes. Let $d_{\omega }^{\text{opt}}$ be the optimal regime for the utility defined by *ω*. Assume that there exists a true utility function defined by some *ω*_0_ = (*ω*_1,0_, …, *ω*_*K*−1,0_) such that observed decisions were made with the intent to maximize *U* = *u*(*y*_1_, *…, y*_*K*_; *ω*_0_). Further assume that treatment decisions in the observed data follow $\text{Pr}\left\{A=d_{\omega_{0}}^{\text{opt}}(x)|X=x\right\}=\text{expit}\left(x^{⊤}\beta \right)$, where *β* is an unknown parameter.

Define $Q_{Y_{k}}(x,a)=E\left(Y_{k}|X=x,A=a\right)$, for *k* = 1, …, *K*. Define also

$$Q_{\omega }(x,a)=E\left\{u\left(Y_{1},\ldots ,Y_{K};\omega \right)|X=x,A=a\right\}$$

and note that $Q_{\omega }(x,a)=\sum_{k=1}^{K-1}\omega_{k}Q_{Y_{k}}(x,a)+\left(1-\sum_{k=1}^{K-1}\omega_{k}\right)Q_{Y_{K}}(x,a)$. The Q-functions for each outcome can be estimated from the observed data using regression models. Let ${\hat{Q}}_{Y_{k},n}(x,a)$ denote an estimator for $Q_{Y_{k}}(x,a)$. Then, an estimator for *Q*_*ω*_(***x***, *a*) is ${\hat{Q}}_{\omega ,n}(x,a)=\sum_{k=1}^{K-1}\omega_{k}{\hat{Q}}_{Y_{k},n}(x,a)+\left(1-\sum_{k=1}^{K-1}\omega_{k}\right){\hat{Q}}_{Y_{K},n}(x,a)$. For any fixed *ω*, we can compute an estimator for $d_{\omega }^{\text{opt}}$ as ${\hat{d}}_{\omega ,n}(x)=\text{arg }{\text{max}}_{a\in A}{\hat{Q}}_{\omega ,n}(x,a)$. The pseudo-likelihood is

(14)
$${\hat{L}}_{n}(\omega ,\beta )\propto \prod_{i=1}^{n}\frac{\text{exp}\left[X_{i}^{⊤}\beta 1\left\{A_{i}={\hat{d}}_{\omega ,n}\left(X_{i}\right)\right\}\right]}{1+\text{exp}\left(X_{i}^{⊤}\beta \right)},$$

for a vector *β* and a vector *ω* = (*ω*_1_, …, *ω*_*K*−1_). For *K* = 2, this reduces to the formulation in Section 2. Estimators for *β* and *ω*_1_, …, *ω*_*K*−1_ can be obtained by maximizing the pseudo-likelihood in (14). Letting ${\hat{\omega }}_{n}=({\hat{\omega }}_{1,n},\ldots ,{\hat{\omega }}_{K-1,n}$ denote the maximum pseudo-likelihood estimator for *ω*, an estimator for the optimal regime is ${\hat{d}}_{{\hat{\omega }}_{n},n}(x)=\text{arg }{\text{max}}_{a\in A}{\hat{Q}}_{{\hat{\omega }}_{n},n}(x,a)$.

When the number of outcomes is large, maximizing (2) using the grid search proposed in Section 2.1 is infeasible. However, we can use the Metropolis algorithm similar to that proposed for a patient-specific utility function. A patient-specific utility function can be accommodated by setting $u\left(y_{1},\ldots ,y_{K};x,\theta \right)=\sum_{k=1}^{K-1}\text{expit}\left(x^{⊤}\theta_{k}\right)y_{1}+\left\{1-\sum_{k=1}^{K-1}\text{expit}\left(x^{⊤}\theta_{k}\right)\right\}y_{K}$ for a vector $\theta ={\left(\theta_{1}^{⊤},\ldots ,\theta_{K-1}^{⊤}\right)}^{⊤}$ and maximizing the pseudo-likelihood using the Metropolis algorithm.

To examine the performance of the proposed method in the presence of more than two outcomes, we use the following generative model. As before, let ***X*** = (*X*_1_, …, *X*_5_)^**⊤**^ be independent normal random variables with mean 0 and standard deviation 0.5. Let *ϵ*_1_, *ϵ*_2_ and *ϵ*_3_ be independent normal random variables with mean 0 and standard deviation 0.5. Given treatment assignment, outcomes are generated according to *Y*_1_ = *A*(4*X*_1_ − 2*X*_2_ + 2) + *ϵ*_1_, *Y*_2_ = *A*(2*X*_1_ − 4*X*_2_ − 2) + *ϵ*_2_, and *Y*_3_ = 1 + *A*(*X*_1_ + *X*_2_ + 1)*ϵ*_3_. For a fixed *ω* = (*ω*_1_, *ω*_2_) and fixed *ρ* ∈ [0, 1], treatment assignment is made according to $\text{Pr}\left\{A=d_{\omega }^{\text{opt}}(x)|X=x\right\}=\rho$.

We set *ω*_1_ = 0.2, *ω*_2_ = 0.4, and *ρ* = 0.6, 0.8. Table 12 contains parameter estimates averaged across 500 replications along with standard deviations (in parentheses) across replications. The error rate is the proportion of samples in a testing set that were assigned the optimal treatment by the estimated policy. Table 13 contains estimated values (calculated by generating an independent testing set following the same generative model but with decisions made according to each policy) of the optimal policy, a policy where the utility function is estimated (the proposed method), policies estimated to maximize each outcome individually, and standard of care. The proposed method results in values close to the true optimal in large samples and larger than maximizing each individual outcome across sample sizes.

**Table 12:** Estimation results for simulations where utility and probability of optimal treatment are fixed, with three outcomes.

*n*	*ρ*	${\hat{\omega }}_{1,n}$	${\hat{\omega }}_{2,n}$	${\hat{\rho }}_{n}$	Error rate
100	0.60	0.21 (0.16)	0.34 (0.20)	0.63 (0.07)	0.15 (0.11)
	0.80	0.21 (0.07)	0.42 (0.09)	0.81 (0.04)	0.04 (0.03)
200	0.60	0.21 (0.13)	0.40 (0.17)	0.62 (0.04)	0.11 (0.09)
	0.80	0.21 (0.04)	0.41 (0.06)	0.80 (0.03)	0.03 (0.02)
300	0.60	0.21 (0.12)	0.39 (0.16)	0.62 (0.03)	0.09 (0.08)
	0.80	0.20 (0.03)	0.41 (0.04)	0.80 (0.02)	0.02 (0.01)
500	0.60	0.21 (0.09)	0.41 (0.12)	0.61 (0.02)	0.06 (0.05)
	0.80	0.20 (0.02)	0.40 (0.03)	0.80 (0.02)	0.01 (0.01)

**Table 13:** Value results for simulations where utility and probability of optimal treatment are fixed, with three outcomes.

*n*	*ρ*	Optimal	Estimated utility	*Y*_1_ only	*Y*_2_ only	*Y*_3_ only	Standard of care
100	0.60	1.38 (0.04)	1.28 (0.15)	1.09 (0.06)	1.00 (0.06)	0.62 (0.10)	0.59 (0.14)
	0.80	1.39 (0.04)	1.37 (0.06)	1.09 (0.06)	1.00 (0.06)	0.62 (0.11)	0.99 (0.13)
200	0.60	1.38 (0.04)	1.32 (0.12)	1.09 (0.06)	1.00 (0.06)	0.62 (0.08)	0.60 (0.10)
	0.80	1.39 (0.04)	1.38 (0.05)	1.09 (0.05)	1.00 (0.06)	0.62 (0.09)	0.98 (0.09)
300	0.60	1.38 (0.04)	1.34 (0.10)	1.09 (0.06)	1.00 (0.06)	0.63 (0.08)	0.60 (0.08)
	0.80	1.38 (0.04)	1.39 (0.05)	1.10 (0.06)	1.00 (0.06)	0.63 (0.08)	0.99 (0.07)
500	0.60	1.39 (0.04)	1.36 (0.07)	1.09 (0.05)	1.00 (0.06)	0.62 (0.07)	0.60 (0.06)
	0.80	1.39 (0.04)	1.38 (0.05)	1.10 (0.06)	1.00 (0.06)	0.63 (0.07)	0.99 (0.06)

## Appendix C: Misspecified Model for the Utility Function

In this section, we demonstrate that the proposed method achieves reasonable performance even in the presence of a misspecified model for the utility function. Let ***X***, *Y*, and *Z* be generated as above. Let the true underlying utility function be *u*(*y, z*; ***x***, *θ*) = *ω*(***x***; *θ*)*y* + {1 − *ω*(***x***; *θ*)}*z*, where $\omega (x;\theta )=\text{expit}\left(1+x_{1}^{2}+x^{⊤}\theta_{0}\right)$ with *θ*_0_ = (−0. 5, 0, 0, 1, 0.5)^**⊤**^. The misspecified model fit to estimate the utility function contained only an intercept, *X*_1_, *X*_2_, *X*_3_, and *X*_4_. Therefore, these simulations represent the setting where one important covariate and a squared term for one covariate are omitted from the model for the utility function. Treatment was assigned according to $\text{Pr}\left\{A=d_{\omega }^{\text{opt}}(x)|X=x\right\}=\text{expit}\left(0.5+x_{1}\right)$. Table 14 contains the estimated value when the model for the utility function is correctly specified and when the model is incorrectly specified, along with the value of the true optimal policy and the standard of care. The proposed method produces comparable results regardless of whether the utility function is misspecified or not.

**Table 14:** First simulation results with a misspecified model for the utility function.

*n*	Optimal	Correct	Misspecified	Standard of Care
100	1.86 (0.07)	1.61 (0.21)	1.64 (0.20)	0.59 (0.23)
200	1.85 (0.07)	1.68 (0.16)	1.69 (0.17)	0.57 (0.16)
300	1.86 (0.07)	1.72 (0.13)	1.74 (0.13)	0.57 (0.13)
500	1.86 (0.07)	1.77 (0.10)	1.76 (0.11)	0.58 (0.10)

Table 15 contains results for the same model misspecification, but where the true utility function is $\omega (x;\theta )=\text{expit}\left(1+4x_{1}^{2}+x^{⊤}\theta_{0}\right)$ with *θ*_0_ = (−0.5, 0, 0, 1, 4)^**⊤**^, i.e., the coefficients for the components that are left out of the misspecified model are larger. When the coefficients of the components left out of the utility function model are larger, the proposed method produces better results when the model is correctly specified. However, even in the presence of model misspecification, the proposed method produces results that improve upon the standard of care.

**Table 15:** Second simulation results with a misspecified model for the utility function.

*n*	Optimal	Correct	Misspecified	Standard of Care
100	2.11 (0.08)	1.63 (0.28)	1.64 (0.23)	0.69 (0.26)
200	2.11 (0.08)	1.76 (0.22)	1.68 (0.18)	0.67 (0.18)
300	2.10 (0.07)	1.84 (0.19)	1.70 (0.16)	0.68 (0.15)
500	2.10 (0.08)	1.88 (0.16)	1.73 (0.15)	0.67 (0.12)

## Appendix D: Misspecified Model for the Probability of Assigning the Optimal Treatment

Similar to the model with the misspecified utility function, the model with the misspecified probability of the optimal treatment resulted in the values that are comparable to the correct model. ***X***, *Y* and *Z* were generated as above. Let $\omega (x;\theta )=\text{expit}\left(1+4x_{1}^{2}+x^{T}\theta_{0}\right)$, with *θ*_0_ = (−0.5, 0, 0, 1, 0.5) and $\text{Pr}\left\{A=d_{\omega }^{\text{opt}}|X=x\right\}=\text{expit}\left(0.5+4x_{1}^{2}+x_{1}\right)$. For this simulation, the misspecified model for the probability of assigning the optimal treatment is assumed to not include $4X_{1}^{2}$. In Table 16, the estimated value of the model with the misspecified probability of assigning the optimal treatment is confirmed to be similar to the estimated value of the correct model.

**Table 16:** Value results with a misspecified model for the probability of assigning the optimal treatment.

*n*	Optimal	Correct	Misspecified	Standard of Care
100	2.01 (0.07)	1.90 (0.14)	1.89 (0.20)	1.29 (0.23)
200	2.01 (0.08)	1.96 (0.09)	1.95 (0.12)	1.29 (0.16)
300	2.01 (0.08)	1.97 (0.09)	1.97 (0.10)	1.29 (0.13)
500	2.00 (0.08)	1.98 (0.08)	1.98 (0.08)	1.30 (0.10)

In the next simulation, let *ω*(***x***; *θ*) = expit(1 + ***x***^T^*θ*_0_) with *θ*_0_ = (−0.5, 0, 0, 1, 0.5) and the true probability of assigning the optimal treatment is $\text{Pr}\left\{A=d_{\omega }^{\text{opt}}(x)|X=x\right\}=\text{expit}\left(0.5+x_{1}\right)$ when the misspecified model is assumed as a constant. In Table 17, it is noticeable that the difference between the two estimated values is similar as in Table 16. For all settings above, the estimated value of the correct model is similar to the estimated value of the models with both the misspecified utility functions and the misspecified probability of optimal treatment.

**Table 17:** Value results with a misspecified model for the utility function.

*n*	Optimal	Correct	Misspecified	Standard of Care
100	1.76 (0.06)	1.66 (0.19)	1.67 (0.19)	0.51 (0.22)
200	1.76 (0.06)	1.71 (0.12)	1.71 (0.13)	0.49 (0.15)
300	1.76 (0.06)	1.72 (0.11)	1.74 (0.08)	0.50 (0.13)
500	1.76 (0.06)	1.75 (0.07)	1.75 (0.06)	0.50 (0.10)

## Appendix E: Relationship between the probability of assigning the optimal treatment and the variance of the estimator of the utility

**Table 18:** Estimates of *ω* across different *ρ*s

*n*	_*ω*_╲^*ρ*^	0.2	0.3	0.35	0.4
100	0.25	0.25 (0.05)	0.25 (0.09)	0.27 (0.15)	0.33 (0.24)
	0.75	0.75 (0.05)	0.75 (0.10)	0.74 (0.14)	0.66 (0.24)
200	0.25	0.25 (0.03)	0.25 (0.05)	0.25 (0.08)	0.28 (0.16)
	0.75	0.75 (0.03)	0.75 (0.05)	0.75 (0.09)	0.73 (0.13)
300	0.25	0.25 (0.02)	0.25 (0.04)	0.25 (0.07)	0.26 (0.12)
	0.75	0.75 (0.02)	0.75 (0.04)	0.75 (0.07)	0.73 (0.13)
500	0.25	0.25 (0.01)	0.25 (0.02)	0.25 (0.04)	0.25 (0.08)
	0.75	0.75 (0.01)	0.75 (0.03)	0.75 (0.05)	0.76 (0.08)

**Table 19:** Estimates of *ω* across different *ρ*s

*n*	_*ω*_╲^*ρ*^	0.6	0.65	0.7	0.8
100	0.25	0.34 (0.24)	0.27 (0.14)	0.25 (0.10)	0.25 (0.05)
	0.75	0.66 (0.24)	0.73 (0.15)	0.74 (0.10)	0.75 (0.05)
200	0.25	0.28 (0.16)	0.25 (0.08)	0.25 (0.05)	0.25 (0.02)
	0.75	0.72 (0.16)	0.75 (0.08)	0.75 (0.05)	0.75 (0.03)
300	0.25	0.26 (0.11)	0.25 (0.07)	0.25 (0.04)	0.25 (0.02)
	0.75	0.74 (0.13)	0.75 (0.07)	0.75 (0.04)	0.75 (0.02)
500	0.25	0.25 (0.08)	0.25 (0.04)	0.25 (0.03)	0.25 (0.01)
	0.75	0.75 (0.08)	0.76 (0.05)	0.75 (0.02)	0.75 (0.01)

In this section, we explore the relationship between the probability of assigning the optimal treatment and the variance of the estimator of the utility, which was mentioned in Remark 15. We empirically examine how the standard error of the estimator of the utility changes as $\text{Pr}\left\{A=d_{\omega }^{\text{opt}}(x)|X=x\right\}$ varies. For simplicity, we focus on the fixed utility setting described in 4.1. Let ***X***, *Y* and *Z* be generated as in 4.1. For each *ρ*s, we estimate $\hat{\omega }$ when *ω* = 0.25 and *ω* = 0.75.

Table 18 and Table 19 display $\hat{\omega }$ and $s.e.\;(\hat{\omega })$ across values of *n*, *ω*, and *ρ*. As *ρ* deviates from 0.5 (so that *β* is not close to 0), $\hat{\omega }$ is closer to the true *ω*, and the standard error of $\hat{\omega }$ decreases. Moreover, if *ω*_1_ and *ω*_2_ satisfy *ω*_1_ = 1 − *ω*_2_, their estimates and standard errors are similar as logit(*ω*_1_) = −logit(*ω*_2_).

## Appendix F: Performance of Metropolis optimizer in the algorithm with the patient-specific utility.

**Table 20:** Absolute difference and the Euclidean distance of the estimates of the patient utility and *θ*_0_

*n*	Absolute diffrence-MH	Absolute difference-Unit ball	Distance-MH	Distance-Unit ball
100	1.49 (0.71)	1.66 (0.30)	0.85 (0.45)	0.86 (0.12)
200	1.30 (0.49)	1.60 (0.31)	0.72 (0.31)	0.84 (0.13)
300	1.19 (0.20)	1.63 (0.29)	0.63 (0.16)	0.86 (0.13)
500	1.15 (0.12)	1.63 (0.32)	0.60 (0.09)	0.85 (0.13)

In this section, we examine the performance of the Metropolis optimizer that is used in 2.2. We randomly select 10000 points in the unit ball around *θ*_0_, and use the point that yields the best likelihood as the reference for the comparison. We define $\hat{\theta }$ by Metropolis algorithm as ${\hat{\theta }}_{\text{MH}}$, and a best point from a unit ball around *θ*_0_ as ${\hat{\theta }}_{\text{Uball }}$. We obtain the distance between *θ*_0_ and ${\hat{\theta }}_{\text{MH}}$ and the distance between *θ*_0_ and ${\hat{\theta }}_{\text{Uball}}$ for each of 500 replications.

In Table 20, absolute difference-MH was calculated as the mean of $\left|{\hat{\theta }}_{\text{MH}}-\theta_{0}\right|$, and absolute difference-Unit ball was calculated as the mean of $\left|{\hat{\theta }}_{\text{Uball }}-\theta_{0}\right|$ for 500 replications. Similarly, Distance-MH was calculated as the mean of $\sqrt{\sum_{j=1}^{6}{\left({\hat{\theta }}_{\text{MH},j}-\theta_{0,j}\right)}^{2}}$, and Distance-Unit ball was calculated as the mean of $\sqrt{\sum_{i=1}^{6}{\left({\hat{\theta }}_{\text{Uball },j}-\theta_{0,j}\right)}^{2}}$ for 500 replications. The standard errors of these distances are in the parentheses.

By Table 20, it is noticeable that both absolute difference and the Euclidean distance of ${\hat{\theta }}_{\text{MH}}$ are smaller, and we could conclude that it is reasonable to use Metropolis algorithm when estimating a patient-specific utility.
